# Deciphering Molecular Aspects of Potential *α*-Glucosidase Inhibitors within *Aspergillus terreus*: A Computational Odyssey of Molecular Docking-Coupled Dynamics Simulations and Pharmacokinetic Profiling

**DOI:** 10.3390/metabo13080942

**Published:** 2023-08-12

**Authors:** Sameh S. Elhady, Noha M. Alshobaki, Mahmoud A. Elfaky, Abdulrahman E. Koshak, Majed Alharbi, Reda F. A. Abdelhameed, Khaled M. Darwish

**Affiliations:** 1 Department of Natural Products, Faculty of Pharmacy, King Abdulaziz University, Jeddah 21589, Saudi Arabia; nhamadaelshoubaki@stu.kau.edu.sa (N.M.A.); melfaky@kau.edu.sa (M.A.E.); aekoshak@kau.edu.sa (A.E.K.); 2 Centre for Artificial Intelligence in Precision Medicines, King Abdulaziz University, Jeddah 21589, Saudi Arabia; 3 Department of Pharmaceutical Chemistry, Faculty of Pharmacy, King Abdulaziz University, Jeddah 21589, Saudi Arabia; maaalharbi1@kau.edu.sa; 4 Department of Pharmacognosy, Faculty of Pharmacy, Galala University, New Galala 43713, Egypt; reda.fouad@gu.edu.eg; 5 Department of Pharmacognosy, Faculty of Pharmacy, Suez Canal University, Ismailia 41522, Egypt; 6 Department of Medicinal Chemistry, Faculty of Pharmacy, Suez Canal University, Ismailia 41522, Egypt

**Keywords:** marine fungi, *Aspergillus terreus*, secondary metabolites, *α*-glucosidase, molecular modeling

## Abstract

Hyperglycemia, as a hallmark of the metabolic malady diabetes mellitus, has been an overwhelming healthcare burden owing to its high rates of comorbidity and mortality, as well as prospective complications affecting different body organs. Available therapeutic agents, with *α*-glucosidase inhibitors as one of their cornerstone arsenal, control stages of broad glycemia while showing definitive characteristics related to their low clinical efficiency and off-target complications. This has propelled the academia and industrial section into discovering novel and safer candidates. Herein, we provided a thorough computational exploration of identifying candidates from the marine-derived *Aspergillus terreus* isolates. Combined structural- and ligand-based approaches using a chemical library of 275 metabolites were adopted for pinpointing promising *α*-glucosidase inhibitors, as well as providing guiding insights for further lead optimization and development. Structure-based virtual screening through escalating precision molecular docking protocol at the α-glucosidase canonical pocket identified 11 promising top-docked hits, with several being superior to the market drug reference, acarbose. Comprehensive ligand-based investigations of these hits’ pharmacokinetics ADME profiles, physiochemical characterizations, and obedience to the gold standard Lipinski’s rule of five, as well as toxicity and mutagenicity profiling, proceeded. Under explicit conditions, a molecular dynamics simulation identified the top-stable metabolites: butyrolactone VI (SK-44), aspulvinone E (SK-55), butyrolactone I 4′’’’-sulfate (SK-72), and terrelumamide B (SK-173). They depicted the highest free binding energies and steadiest thermodynamic behavior. Moreover, great structural insights have been revealed, including the advent of an aromatic scaffold-based interaction for ligand–target complex stability. The significance of introducing balanced hydrophobic/polar moieties, like triazole and other bioisosteres of carboxylic acid, has been highlighted across docking, ADME/Tox profiling, and molecular dynamics studies for maximizing binding interactions while assuring safety and optimal pharmacokinetics for targeting the intestinal-localized α-glucosidase enzyme. Overall, this study provided valuable starting points for developing new α-glucosidase inhibitors based on nature-derived unique scaffolds, as well as guidance for prospective lead optimization and development within future pre-clinical and clinical investigations.

## 1. Introduction

Hyperglycemia can lead, over time, to serious detrimental damage to several body organs as a result of being the common sequel to uncontrolled diabetes mellitus [1]. With particular concern regarding blood vessels and nerves, elevated sugar blood levels could cause atherosclerosis and neuropathy, in addition to other significant complications, such as kidney damage, retinopathy, and dyslipidemia [2]. Within recent years, the metabolic disorder of diabetes mellitus has been categorized as a worldwide healthcare burden owing to its high rates of comorbidity and mortality. The global number of adults afflicted by the disease is expected to increase from 537 million to 643 million individuals by the year 2030, leading to enormous disease-associated economic burdens [3]. Type-II of the disease comprises 95% of the cases, being linked to insulin-secretion deficiencies, cellular-insulin resistances, or a combination [4]. To date, the disease is without a cure, with only available therapies, including insulin shots and oral hypoglycemic agents, being designated for overcoming stages of broad glycemia [2]. Within the modern research of drug discovery, targeting enzymes related to specific disorders for inhibition has been considered a relevant approach [5].

One of the most important biotargets within the human intestine brush borders mucosa is the α-glucosidase enzyme (EC 3.2.1.20), responsible for disaccharides digestion (α−1,4-glycosidic bond hydrolysis) into respective monomeric units prior to gut absorption [6,7]. FDA-approved α-glucosidase inhibitors named acarbose, miglitol, and voglibose have been therapeutically recommended for type-II diabetes mellitus for reducing intestinal carbohydrate hydrolysis and allowing for the control of post-prandial hyperglycemia [8]. Acarbose is, by far, the medication being highly prescribed in cases when therapeutic goals are being unmet or there are contraindications to other oral hypoglycemic agents [9,10,11]. Unfortunately, the long-term usage of these inhibitors is usually associated with mild-to-moderate undesirable gastrointestinal complications, including flatulence, abdominal discomfort, and diarrhea. The latter side effects are mostly related to the off-target inhibition of α-amylase, rather than α-glucosidase causing increased sections of undigested starch and glycogen within the large intestine [12]. Thus, developing potent selective α-glucosidase inhibitors would be considered safe and more tolerable. Additionally, developing such compounds would be also beneficial for managing obesity and other α-glucosidase-associated diseases, including viral infections, malignancy, and α-glucosidase-related maladies [13,14,15,16]. On the pharmaceutical industry bases, the synthesis of FDA-approved drugs has been introduced through multistep, cost-ineffective, complicated biosynthetic pathways, owing to their sugar-based chemical structures [17,18]. Therefore, introducing novel scaffolds with straightforward synthetic/semisynthetic pathways, as well as improved pharmacodynamic/pharmacokinetic profiles, would be valuable for patient compliance and disease management.

The pipeline discovery of new compounds has been long hampered by traditional drug-discovery and -development processes being time inefficient, expensive, and laborious [19]. Recent advances within bioinformatics and cheminformatics, as well as improved algorithms and software, have settled computational approaches as indispensable tools for increasing the efficacy of new drug discovery and development as they rely on molecular modeling [20]. Mimicking target–target or ligand–target interactions, predicting pharmacokinetics/toxicity parameters, and undertaking better experimental planning through guiding/limiting in vivo/in vitro tests have all reduced both time and expenses [19]. These benefits could increase the population’s access to medicine by reducing the cost of goods [20]. Within such context, computational tools of virtual screening become promising within the drug discovery and development process. The milestone of virtual screening was the market introduction of several drugs, including the anticancer agent gefitinib, being recognized through screening 1500 compounds using the ALLADIN platform [21]. Other drugs that reached the market with the assistance of molecular modeling include the anti-hypertensive agents captopril and aliskiren; several anti-HIV agents (indinavir, saquinavir, and ritonavir); a selective anti-influenza agent (zanamivir); dorzolamide, for managing glaucoma; hepatitis C viral protease inhibitor (boceprevir); and phase-III clinical trial nolatrexed, for managing liver cancer [22,23]. Other than the tools-of-trade, molecular modeling has made the most significant advent of drug resources for identifying successful compounds targeting biotargets for disease management.

Nature has been considered an enteral source of novel and structurally diverse compounds of both fascinating and, in most cases, complex chemical scaffolds. Driven by exploring uncharted arenas, there has been a rise in interest in the isolation of α-glucosidase inhibitors from particular bacteria. For instance, the new *N*-containing malto-oligosaccharide, GIB-638, was isolated from a culture filtrate of *Streptomyces fradiae* PWH638; validamycin A was isolated from *Streptomyces hygroscopicus* var. limoneus, a broth of *Bacillus subtilis* B2 also possessed strong α-glucosidase activity, and aspergillusol A was isolated from the marine-derived fungus *Aspergillus aculeatus*. Therefore, research is still required to explore potential novel α-glucosidase inhibitors to guide future medication development [24]. *Aspergillus terreus* was originally studied in 1918, with its ubiquitous soil saprophyte subject to a global distribution. It was isolated from both marine and terrestrial sources. Lovastatin, a statin medication that inhibits 3-hydroxy-3-methylglutarylcoenzyme and one of the FDA-approved chemical entities for treating hyperlipidemia and managing coronary heart diseases, has been identified as a secondary metabolite of *A. terreus* [25]. Notably, the species *A. terreus* is also a primary producer of reductase, which is known for its ability to decrease cholesterol and is used to treat atherosclerosis and heart disease [26]. Recently, aspulvinone E and butyrolactone I, as well as their analogs, were extracted from an ethyl acetate extract of *A. terreus*; we previously reported that they may have potential inhibitory activity against α-glucosidase [27]. As part of our research program on the utilization of bioresources, we are virtually screening α-glucosidase inhibitors derived from *A. terreus* to assess the structure–activity relationship.

The promising anti-diabetic activity of the marine fungus *A. terreus*, as well as the current advances within the computational tools used for highly sophisticated and reliable screening approaches, has propelled us to investigate the fungus-isolated metabolites used for novel drug discovery and guidance toward prospective lead optimization and development. Using a chemical library of literature reporting on *A. terreus-*isolated metabolites, we conducted a multi-stage structure-based virtual screening through escalating precision molecular docking protocol at the α-glucosidase canonical pocket. Employing ligand-based approaches for the 11 top-docked hits proceeded throughout the comprehensive investigation of the hits’ pharmacokinetics profiles, physiochemical characterization, and obedience to the gold standard of Lipinski’s rule of five, in addition to both toxicity and mutagenicity studies. Finally, the thermodynamic behaviors of these 11 hits, as compared to the reference market drug, were evaluated through a 200 ns explicit molecular dynamic simulation used for validating their complex stability and providing valuable insights guiding future candidate development and optimization.

## 2. Materials and Methods

### 2.1. Structure Preparation

The atomic structure of the human intestinal maltase-glucoamylase *α*-glucosidase enzyme (*hi*MGAM) was sourced from the RCSB Protein Data Bank (PDB) with an entry ID of 2QMJ (https://www.rcsb.org/structure/2QMJ; (access on 24 November 2022)) [28]. The deposited structure comprises 870 total residues within an asymmetric monomeric-A1 chain, being solved in terms of an X-ray crystallographic technique applied at a high-resolution index of 1.90 Å and bounded to the non-hydrolyzable target inhibitor, acarbose. The downloaded protein was structurally prepared using the AutoDock Vina tool package v1.2.0 (Scripps Research Institute, La Jolla, CA, USA) by adding the polar and non-polar hydrogens missing from the X-ray crystallized PDB file, as well as assigning the Gasteiger partial charges of the whole protein [29]. Co-crystallized water/solvent/ion molecules were stripped out of the structure to permit the free docking of new ligands within the target’s active site. The prepared target was converted into a pdbqt.file extension to be saved for later usage.

The compound library was constructed using our preliminary survey and was used for identifying *A. terrus-*isolated compounds, which finally comprised the 276 identified structurally diverse chemical isolates. These compounds were extracted from 3571 journal articles spanning 286 journal titles. Identified metabolites were finally crosschecked through open-access databases: the Collection of Open Natural Products (COCONUT; https://coconut.naturalproducts.net/; (access on 25 November 2022)) and PubChem (https://pubchem.ncbi.nlm.nih.gov/; (access on 28 November 2022)). All ligands were built and converted into 3D-structural fashions via the ChemDraw/ChemBioOffice v19.1 package (PerkinElmer, Waltham, MA, USA), relying on the ligands’ respective isomeric SMILES strings obtained from the PubChem chemical library. Compounds were then energy minimized under a hybrid forcefield combining empirical and knowledge-based functions at pH 7.4 using the AutoDock tool package, where the forcefield is well parameterized for the gas-phase small organic molecules of the medicinal chemistry during strong performances [30]. The prepared and minimized ligands were finally converted into pdbqt.files, using the OpenBabel tool v.2.3.1 (National Supercomputer Centre, Linköping, Sweden) [31] for their subsequent molecular docking virtual screening at the *hi*MGAM target protein.

### 2.2. Molecular Docking-Driven Virtual Screening

Virtual screening for the 265 natural isolates proceeded through a multi-stage molecular docking simulation on the AutoDock Vina 1.2.0 platform, applying the Lamarckian genetic algorithm and empirical/knowledge-based hybrid scoring function under flexible ligand–rigid receptor docking protocol [32]. The initial filtering stage involved a rapid structure-based virtual screening protocol with reasonable computational expenditure for the whole 276 metabolites, with the exhaustiveness parameter at AutoDock vina being set at a value of 8. Later, the filtered compounds were redocked under two sequentially higher exhaustiveness values of 32 and 50 for obtaining the final best-docked compounds of the best-predicted biological activity regarding the most consistent docking findings [33]. The computational workflow was conducted on a Dell^®^ Precision 7920 (Dell Technologies, Irvine, CA, USA) equipped with Intel^®^ Xeon-Gold 5220R 4.0 GHz Turbo and Dual Nvidia^®^ Quadro-RTX 5000, 16GB professional graphics and VirtualLink (XX-20T).

Setting the binding site for docking was guided by the information obtained from the co-crystallized ligand bounded to the *hi*MGAG target protein. A grid docking box was created to accommodate all essential amino acids being viewed at the deposited complex file, as well as those being reported as crucial for the anchoring of small molecule ligands [28,34,35,36]. Using the grid generation tool within AutoDock, a grid box was constructed at the center atom of the co-crystallized ligand, with an external size of 80 Å × 80 Å × 80 Å and 0.38 Å grid-point spacing along the XYZ-cartesian coordinates. Catalytic residues, as well as polar and non-polar amino acids within proximal contact to acarbose, were all considered during the grid box construction. The grid box included the following pocket-lining residues: Arg202–Asn207, Asn209, Thr211, Tyr214, Arg298, Tyr299, Asp327, Ile328, Ile364, Trp441, Asp443, Met444, Ser448, Arg526, Trp539, Gly541, Asp542, Asp571, Phe575–Leu577, Arg598, His600, Gly602, Gln603, Phe605, Val405, Trp406, Ser448, Phe450, Leu473, and Asp474.

The selection of the best docking pose for promising lead compounds was considered based on furnishing high dock binding energies, mean deviation values (RMSDs) below a 2.0 Å cut-off in relation to the co-crystalline ligand, and significant interactions with reported crucial pocket residues. The visual inspection and protein–ligand interaction analysis for the furnished docking poses were conducted via PyMol2.0.6 Graphical Visualization Software (Schrödinger^TM^, New York City, NY, USA). Validation of the docking protocol was conducted through the redocking approach of the co-crystallized ligand under similar docking conditions (exhaustiveness value 50) being adopted for the top-docked investigated metabolites [37,38,39,40].

### 2.3. Molecular Dynamics Simulations

Exploring the thermodynamic nature and stability of the hit–protein complexes proceeded through molecular dynamics simulations using the GROMACS-2019 software package under the CHARMM-36m ForceField [41,42]. Parameterization and topology files of the investigated hits were automatically generated via the CHARMM-General ForceField program Param-Chem project [43]. Models were individually solvated in cubic boxes of TIP3P under periodic boundary conditions at 10 Å minimum marginal distances [44]. Residues underwent standard ionization under physiological pH (7.4) and entire systems were neutralized via potassium chloride ions via the Monte-Carlo algorithm [45]. The steepest descent minimization (5 ps) [46] and subsequent double equilibration stages (NVT and NPT ensembles, 100 ps foreach stage) were performed under constant force (1000 kJ/mol·nm^2^) [47], maintaining the original protein fold and heavy atom restraining. Production proceeded for 200 ns under the NPT ensemble and the particle-mesh Ewald computed the long-range electrostatic interactions and LINCS for modeling all covalent bonds [48,49]. Van der Waals forces and Coulomb’s non-bonded interactions were truncated at 10 Å under the Verlet cut-off scheme [50]. Root-mean-square deviation (RMSD) and RMS fluctuation (RMSF) were monitored across the whole of the simulation trajectories. The molecular mechanics_Poisson–Boltzmann surface area (MM_PBSA) calculation estimated the ligand’s binding-free energy based on the following equation [51]:

Δ*E*_binding_ = *E*_complex−_(*E*_ligand_ + *E*_target_)

Δ*E_molecular entity_* = *E*_bonded_ + (*E*_van der Waals_ + *E*_electrostatic_)−*TS* + *E*_polar Poisson-Boltzmann equation_ + *γSASA* + *b*

where, *E*_X_ is the total free energy of the target, ligand, or ligand–target complex; *TS* is an entropic contribution to free energy; *γ* and *b* are, respectively, surface tension and fitting constants; and SASA is solvent-accessible surface area. Representation of the ligand–protein conformations was furnished using the PyMol2.0.6 software.

## 3. Results and Discussion

### 3.1. Molecular Docking Analysis

**Figure 1:**
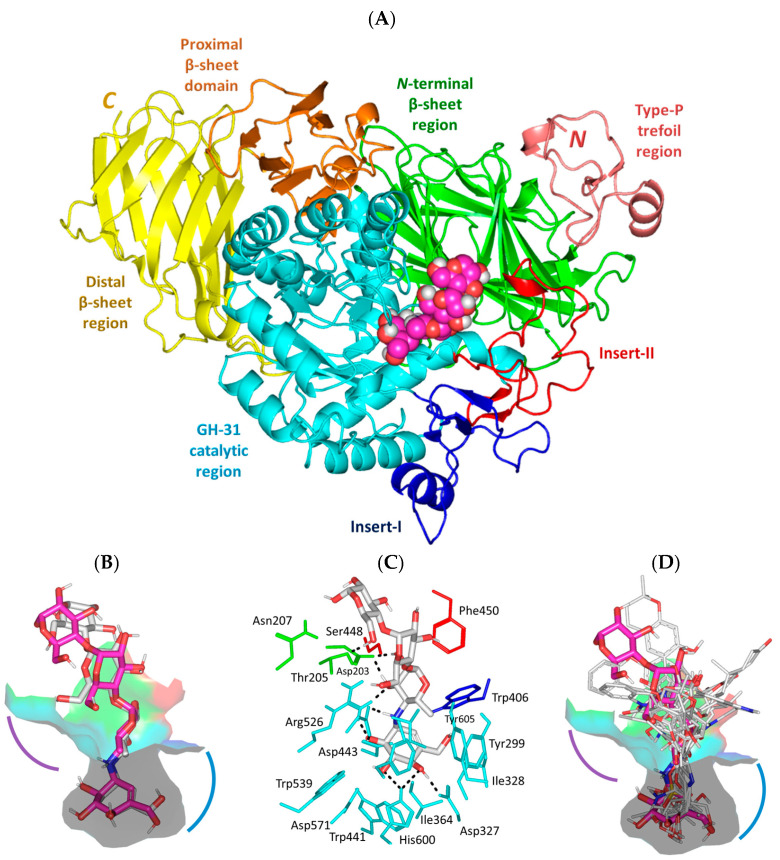
Structure of *hi*MGAM (PDB ID: 2QMJ) bounded with acarbose co-crystalline and investigated marine-based molecules. (**A**) Cartoon representation of *hi*MGAM crystallized with acarbose (magenta spheres) illustrating structural regions within different colors: *N*-terminal P-type trefoil region (deep salmon; Val7–Ser51), *β*-sheet sandwiched region (green; Tyr52–Thr269), catalytic [*α*/*β*]_8_-barrel region (cyan; Pro270–Val651). Comprising insert-I (blue; Pro367–Thr416) and insert-II (red; Val447–Lys492), *C*-terminal proximal region (orange; Ala652–Arg730), and the distal region (yellow; Gly731–His870). (**B**) Overlayed binding modes of redocked (gray sticks) and crystallized acarbose (magenta sticks) at the shallow substrate-binding pocket. (**C**) Binding mode of redocked acarbose; residues located within a 4 Å radius of bound ligand are displayed as lines, numbered with their sequence at the protein, and colored based on the respective domain location. Polar interactions (hydrogen bonding) are shown as black dashed lines. (**D**) Overlayed binding modes of docked compounds (gray lines) and crystallized acarbose (magenta sticks) at the shallow substrate-binding pocket. Both the −1 and +1 carbohydrate subsites are displayed as arcs in, respective, blue and purple colors.

Reported *α*-glucosidase activities regarding several *A. terrus*-isolated metabolites within our literature review [52,53,54,55,56,57,58,59] have prompted us to further investigate the potentiality of identifying novel uncited hits with potential biological activity. Additionally, exploring the molecular aspects of the compound’s affinity/binding with human *α*-glucosidase biological targets through a sophisticated in silico study was rationalized in order to guide future lead optimization and development. The adopted *hi*MGAM biotarget comprises a unique architecture of the *N*-terminal trefoil P-type domain being followed by sandwiched *β*-sheet and catalytic [*α*/*β*]_8_-barrel domains, where the latter bears two inserts, namely, insert-I and insert-II, that arise after respective *β*3 and *β*4 sheets (Figure 1A). At the *C*-terminal, distinct *β*-sandwiched proximal and distal regions were depicted as being comparable to the closely related glycoside hydrolase-31 family [60,61,62,63]. The substrate-binding site, which was adopted here as the canonical docking pocket, mostly involves the residues of the [*α*/*β*]_8_-barrel catalytic domain, as well as those at the *N*-terminus loop (Pro200–Leu217), and portions of catalytic inserts-I/II being proximal to the [*α*/*β*]_8_-barrel opening.

The adopted molecular docking protocol was confirmed as highly valid since a low redocking RMSD value (1.585 Å) was obtained for the redocked co-crystalline ligand, acarbose, in relation to its reference orientation/conformation within the crystallized complex. Depicting RMSD values of less than 2.0 Å signifies that both the adopted docking algorithms and parameters were efficient for predicting relevant binding poses, the thing that would ensure their respective biological significance and, in turn, the docking energies [37]. Performing a flexible ligand–rigid receptor docking workflow within the presented study by AutoDock Vina, rather than flexible receptor mode, was highly reasoned. Preliminary analysis regarding the impact of ligand binding on target conformation was conducted where superposition correlation analysis between the protein’s holo and apo states (PDB ID: 2QMJ and 2QLY, respectively) illustrated non-significant conformational changes (RMSD_Holo aligned Apo_ = 0.131 Å), either locally or globally. Universal protein’s stability on ligand anchoring was further confirmed through data from the reported literature and B-factor analysis, as well as having a shallow solvent-exposed substrate-binding site with possible accommodation of two carbohydrate units [28,60,61]. All of these conferred the negligible impact of local ligand induced-fitting on *hi*MGAM’s ternary protein structure, at least at its macromolecular crystalline states [64].

The redocked protocol managed to replicate the ligand’s crystallized binding orientation/conformation where the acarbose’s initial double rings (i.e., non-reducing acarvosine glycan unit) were anchored at the substrate’s binding-site pocket (Figure 1B). This pseudo-non-reducible disaccharide scaffold depicted a wide range of polar interactions, with Asp203, Asp327, Arg526, Asp542, and His600 sidechains comprising the pocket’s lining residues at −1 and +1 carbohydrate subsites (Figure 1C). Additional water-mediated hydrogen bonding towards Asp443 and Asp571 was also depicted for the acarvosine unit. The acarbose’s initial unit, valienamine aglycone, adopted the ^2^H_3_-half-chair conformation at the −1 subsite, allowing the direction of non-hydrolyzable inter-glycosidic nitrogen atom towards the target’s Asp542 sidechain to be used for hydrogen bond pairing. Both Asp443 and Asp542 are the catalytic residues of *hi*MGAM based on substrate-trapping studies, mutagenesis studies, and sequence-based studies, with GH-31 targets serving as the catalytic nucleophile and acid/base amino acids, respectively. Targeting both residues would halt the hydrolase catalytic machinery of *hi*MGAM [62]. At the +1 subunit pocket, acarbose was stabilized by its C2 and C3 hydroxyl groups mediating polar interactions with Arg526, Asp542, and Asp203 at the *N*-terminal *β*-sheet region. Interestingly, loop residues at the *N*-terminus *β* sheet were reported with invariable interactions with several carbohydrate-based ligands at all resolved GH-31 crystalline structures [60,61,62,63,65]. Regarding the acarbose’s terminal maltose units (+2 and +3 carbohydrate subunits), limited polar interactions with the lining residues were depicted. These terminal scaffolds were generally stabilized via crystal lattice packing and hydrogen bonding for +3 maltose with Thr205 and Asn207, as well as water-bridge polar contacts between +2 maltose and Tyr605 at the pocket’s rim. Further acarbose–protein stability was provided via non-polar contacts with hydrophobic residues: Tyr299, Ile328, Ile364, Trp406, Trp441, Phe450, Trp539, Phe575, Ala576, Leu577, and Tyr605.

The structure-based virtual screening stage involved the multi-staged screening of 265 compounds (chemical library; Supporting Information Table S1) with uprising exhaustiveness values and a 35% top-docking score selection criteria at each screening phase. Exhaustiveness is an AutoDock Vina parameter controlling computational expenditures during docking experiments by setting the numbers of independent runs starting from random conformations of the docked ligands [32]. Smith et al. showed that an exhaustiveness value of 8 is quite recommended for the initial screening stages providing moderate docking power (median RMSD from reference crystallographic structure) with cost-effective computational expenses; however, the findings are likely to be distant from reality [33]. On the other hand, the authors showed higher docking powers (lower median RMSDs) as the adopted exhaustiveness increased; yet, this was at the expense of the computational resources. Little docking power improvements were depicted at exhaustiveness beyond 50, where median RMSDs were kept the same at exhaustiveness values of 75 and 100. In these regards, we decided to perform three sequential docking protocols at respective exhaustiveness values of 8, 32, or 50 while adopting 35% top-docking score selection criteria at each stage for obtaining the final best-docked compounds of the best-predicted biological activity at the most consistent docking findings. The selection percentage was suggested as rational regarding the starting number of compounds for screening, as well as the reported sole success rate with AutoDock Vina [32,66,67]. Out of the initial 265 compounds in the first phase, 94 resulted compounds were passed to the next screening stage. Usingexhaustiveness values under 32 (medium precision) and another 35% selection filter, 33 top-docked compounds were selected for the final screening phase (high precision). A final pool of 11 superior-docked unique compounds was identified as containing relevant hits with maximum possibilities regarding their affinity towards the *hi*MGAM biotarget. None of the identified compounds within the final bin showed less negative docking scores than −8.00 Kcal/mol (Table 1). The highest and lowest docking scores (−11.79 Kcal/mol and –8.75 Kcal/mol, respectively) were assigned for aspulvinone E (SK-55) and aspergillamide A (SK-27), the respective vinylfuranone-based and tripeptide metabolites.

Moving towards the binding modes of the 11 hits, all ligands depicted favored anchoring at the *hi*MGAM’s substrate pocket (Figure 1D). The polar hydrogen bond warhead within the docked compounds predicted deep insertion within the –1 carbohydrate subpocket; meanwhile, the rest of the molecules were directed outwards, reaching towards the +2 and/or +3 subpockets. These depicted binding poses resemble a bunch of flowers emerging from a glass vase. Interestingly, the docked compounds depicted significant overlay over the characteristic conformation/orientation profile of the co-crystallized ligand. Terminal scaffolds of short-structure ligands, including lovastatin (SK-25), aspergillamide A (SK-27), butyrolactone VI (SK-44), aspulvinone E (SK-55), rubrolide S (SK-61), butyrolactone I 4′′′′-sulfate (SK-72), (*+*)-asperteretone F (SK-119), and terrelumamide B (SK-173), managed to only extend towards at the +2 carbohydrate subpocket showing relevant superimpose with the first maltose ring of the acarbose molecule. On the other hand, compounds with more extended structures, such as aspulvinone F (SK-58), 12,15,25,28-tetrahydroxyergosta-4,6,8(14),22-tetraen-3-one (SK-132), and cytochalasin Z11 (SK-182), depicted favored stretching of their terminal moieties towards the terminal acarbose’s maltose ring at the +3 carbohydrate subpocket. Owing to the solvent-exposed nature of the terminal subpockets, lateral substitutions on the compounds’ core skeletons were freely oriented in a way that allowed minimal potential steric hindrances toward the target’s surface. The latter could be translated into favored ligand binding with potential high-affinity/binding energy profiles. It was firstly suggested that an extended ligand would be assigned for higher docking scores owing to their large binding surface of contacts, mostly guided via hydrophobic forces. However, several small-sized compounds (SK-44, SK-55, and SK-72) managed to secure their place as highly favored docked compounds. The latter differential docking scores highlight the significant role of polar contacts with pocket-lining residues on the overall docking scores.

**Figure 2:**
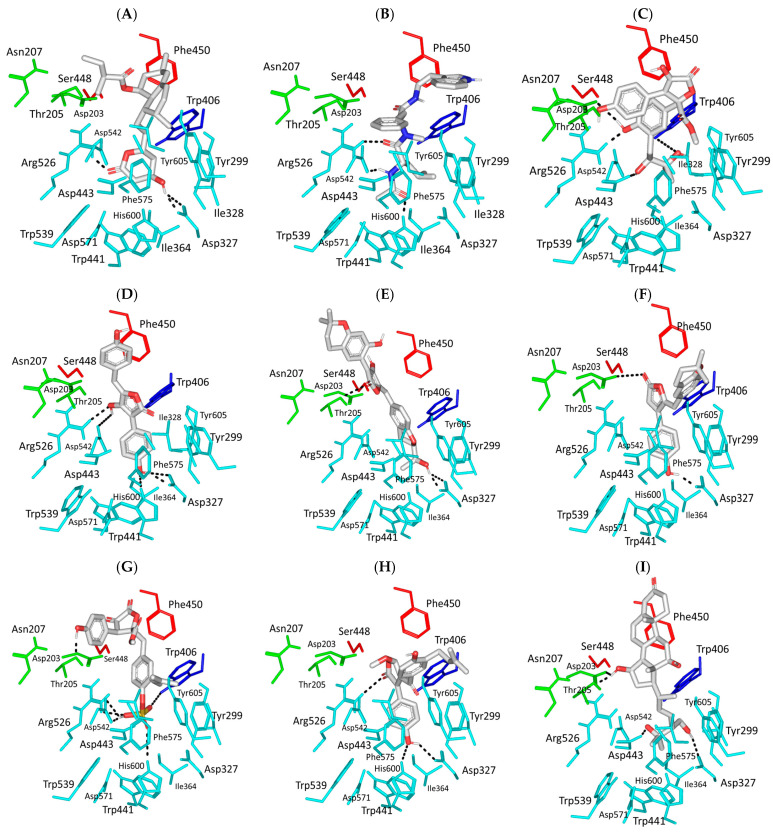

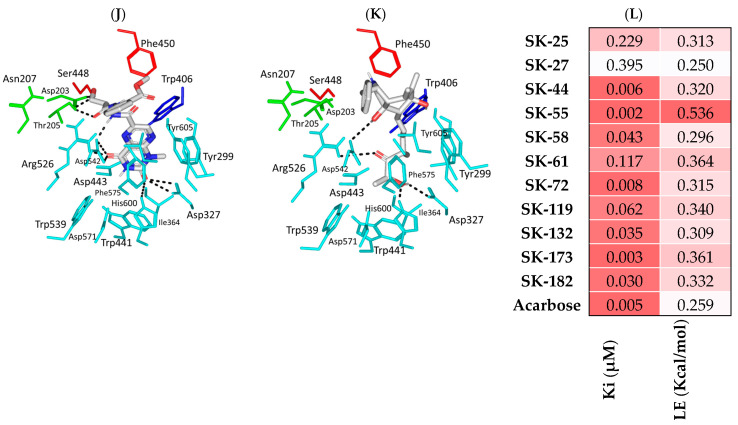
Predicted binding modes of docked *Asperigellus terreus*-isolated compounds at the *h*MGAM binding site: (**A**) lovastatin (SK-25), (**B**) aspergillamide A (SK-27), (**C**) butyrolactone VI (SK-44), (**D**) aspulvinone E (SK-55), (**E**) aspulvinone F (SK-58), (**F**) rubrolide S (SK-61), (**G**) butyrolactone I 4′′′′-sulfate (SK-72), (**H**) (+)-asperteretone F (SK-119), (**I**) 12,15,25,28-tetrahydroxyergosta-4,6,8(14),22-tetraen-3-one (SK-132), (**J**) terrelumamide B (SK-173), (**K**) cytochalasin Z11 (SK-182). Residues located within a 4 Å radius of the bound ligand are displayed as lines, numbered with their sequence at the protein, and colored based on the respective domain location. Polar interactions (hydrogen bonding) are shown as black dashed lines. (**L**) Representing predicted inhibition constant (*Ki*) with ligand’s efficiency (*LE*) of the top-docked identified hits. Heat maps shift darker towards the higher affinity ligands (lower μM concentrations) and most likely predicted hits (higher LE values).

Comprehensive residue-wise ligand/target interaction analysis showed wide-range polar interactions for the top-docked compounds (Table 1 and Figure 2). Both SK-55 and SK-173 depicted the most extended hydrogen bonding, with pocket residues including Asp327 and His600 of the –1 carbohydrate subpocket, as well as Arg526 and catalytic Asp542 within the +1 subsite. Additional stability was granted for these two compounds through extended hydrophobic π-mediated interactions towards Tyr299, Trp406, Phe450, and/or Phe575 at close distances ≤ 5.00 Å. Both polar and non-polar interactions would determine the high predicted docking-binding energies of both compounds (–11.789 and –11.565 Kcal/mol) being even superior to that obtained for the acarbose co-crystallized ligand (–11.388 Kcal/mol). Other ligands, such as SK-25, SK-58, SK-61, SK-119, SK-132, and SK-182, managed to achieve deep anchoring at −1 carbohydrate subsite, furnishing polar contacts with catalytic Asp327 ± His600. The latter observation was suggested for the almost-linear conformation of these ligands’ polar heads, where steric clashes with surrounding pocket residues were limited, allowing the heads’ deep insertions. On the other hand, other ligands exhibited bulkier substitutions, which could not enable proper orientation for these ligands’ polar heads to achieve relevant contacts with catalytic residues at –1 subsite. In return, only the more-linear ligands achieved relevant polar contacts with +1, +2, and even +3 carbohydrate subpockets. This was obvious with SK-44 and SK-72 predicting polar contacts with Asp203, Thr205, and/or Trp406 sidechains. On the contrary, the tripeptide ligand SK-27 failed to achieve such a polar contact pattern and, thus, was assigned with fair docking-binding energy (–8.747 Kcal/mol).

Accumulated evidence from GH-31 family homologs and lysosomal α-glucosidase crystalline structures showed that paucity is mainly associated with the –1 and +1 subsites; whereas, productive substrate-binding sites had quite a moderate impact on +3 subsite residues on ligand’s inhibition profiles [60,61,62,63,65]. However, we suggested that binding to these +3 carbohydrate subsite residues could, to some extent, compensate for the ligand’s retraction-docking mode from the −1 subsite since relatively high docking scores are still achieved with these ligands. Another good binding scenario was seen with lengthy highly extended structured compounds, such as SK-58, SK-132, SK-72, and SK-173, where they managed to stretch across the –1 subsite and up to the +3 subsite, achieving polar contacts with several subsite residues. Other than the polar contacts, the hydrophobic contacts with investigated ligands were almost consistent, including non-polar interactions with Tyr299, Ile328, Ile364, Trp406, Trp441, Met444, Phe450, Leu473, Trp539, Phe575, Ala576, Leu577, and/or Tyr605. Nevertheless, the π-mediated hydrophobic interactions were differential among the docked ligands, where aromaticity within the ligand’s architecture was crucial for such binding. Incorporating aromatic/heterocyclic scaffolds was beneficial for depicting several π–π or π–H contacts that boosted ligand-stabilized binding being observed with several compounds. On the opposite side, lacking aromaticity, as with lovastatin and an ergosterol-based metabolite, was translated to moderate docking-binding energies. Briefing all docking findings has led to suggesting a prospective strategy for structural optimization and structure pharmacophore for better *hi*MGAM binding, where optimal ligands are those with extended architecture and linear polar heads, allowing deep insertion near the catalytic residues. Aromaticity in ligands would add extra stability trade and a competitive advantage over the natural substrates, other than being beneficial in balancing the ligand’s hydrophilic/hydrophobic profile for improved pharmacokinetics/safety profiles [68].

Further bioactivity and lead potentiality assessments proceeded through estimating metricscomprising pharmacodynamic indices with/without molecular descriptors (Figure 2L). The predicted ligand–target inhibition constant (*Ki*) was estimated, relying on the obtained Autodock Vina binding energies (*Ki* = 10^binding energy÷1.366^), and lower *Ki* values down to micromolar ranges were considered relevant for hit/lead consideration [69]. Identified compounds were predicted with low/sub-micromolar activities, with *Ki* values ranging between 0.002 and 0.395 μM concentrations [70]. Other derivable metrics regarding ligand efficiency (*LE*) were estimated within the equation (*LE* = −binding energy ÷ number of heavy atoms), where qualified hits were reported beyond a threshold cut-off at 0.30 [71,72]. All of the natural-isolated metabolites identified from the virtual screening approach depicted values that surpassed the cut-off value, the thing that confers their suitability for being relevant hits that are worth further investigation.

### 3.2. Pharmacokinetics Profiling and Biological-Activity Prediction

The compound’s pharmacokinetic characteristics and safety profiles have been considered the main barriers against the success of candidate drugs throughout the phase-II clinical trials. In these regards, the potentiality of the 11 obtained hits to serve as relevant leads and prospective clinical candidates were evaluated by determining their respective ADME/Tox profiles and drug-likeness indices using QikProp V3.5 (Schrödinger, NY, USA) and TEST V4.2.1 (Toxicity Estimation Software Tool; Environmental Protection Agency, Pennsylvania, DC, USA) [73]. Several physical and pharmaceutical-relevant descriptors were offered by QikProp (Table 2) [74,75,76,77,78] while the compound’s toxicity/safety profiles were further evaluated through the AMES/mutagenicity test and the rat’s oral lethal dose 50 (LD_50_) calculations provided by TEST software. Almost all identified hits were considered promising clinical candidates with fewer potential attritions throughout prospective clinical trials that obeyed the famous Lipinski’s rule of five (RO’5) as the gold standard for clinical candidate success [79,80,81,82]. Moving towards the ADME predicted values, the reference ligand experienced a high polar index (PlogS –2.13) and minimal lipophilicity characteristics (PlogP –5.51), the thing that was translated into poor gut membrane permeations (PPCaco 0.05 nm/sec; 0% oral bioavailability). The findings of this pseudo-polysaccharide drug were inconsistent with the acarbose’s reported experimental values [83]. Depicting such indices is highly rationalized since the drug should concentrate within the gut cellular compartment rather than being absorbed into the blood circulation for a mediating systemic effect. Achieving high drug concentrations at the gut cellular/brush border compartment is considered beneficial for exerting focused inhibition activity on the α-glucosidase enzymes and encountering glucose absorption. Comparable polar/hydrophobic profiles were depicted via the identified hits, SK-72 and SK-173, where negative-valued hydrophobic indices were associated with poor oral bioavailability values (39% and 40%, respectively), as well as gut-membrane permeation. Increased lipophilic characters with values (PlogP from 1.00 to 2.50), as seen with SK-44, SK-55, SK-132, and SK-182, were translated into improved oral bioavailability (~ 80%) and gut–membrane permeation. The most lipophilic hits, SK-25, SK-27, SK-58, and SK-119, were assigned with the highest oral-absorption profiles.

Notably, values of PPMDCK modeling blood–brain barrier permeation were also modest for the highly polar/poor hydrophobic hits (SK-72, SK-132, SK-173, SK182), the things that conferred minimal impact on the CNS compartment. Predicted reduced CNS side effects were also observed through the estimated PlogBB permeations (high negative values up to –3.00). Further safety analysis was highlighted for SK-25, SK-27, SK-44, SK-55, Sk-72, SK-132, and SK-182 through low association with the plasma protein (PlogK_HSA_) and blocking the cardiac HERG_Kv11.1 channels (PlogHERG) predicting values as preference. On the contrary, other hits predicted potential cardiotoxic activity (PlogHERG > –5.0); yet, this was not considered alarming for SK-173 where poor oral bioavailability profiles were assigned. The same latter oral bioavailability/PlogHERG pattern was observed with the reference ligand, the thing that ensured the safety of both SK-173 and acarbose in relation to reported experiments. Combined safety profiles for the above-described ligand were further highlighted through the estimated AMES/mutagenicity test and use of the oral lethal dose 50 (LD_50_) on rats, where the latter represents the compound’s concentration (mg/kg) required for 50% rat death following per-oral administration or the positive induction of colony growth with any *Salmonella typhimurium* strain [84]. The highly polar/poor hydrophobic hits were assigned with the highest dose concentrations required for killing half of the rats (1033.63 to 2008.27 mg/kg). Negative mutagenicity was depicted for all of the investigated hits. Based on the above predicted kinetic findings, increasing the compound’s polarity, as well as lowering hydrophobic characteristics, would be an advent for gut brush border concentration and minimal toxicity profiles. Ligand hits, such as SK-72, SK-173, and SK-182, were the best fitting regarding the profile. For the structural optimization of other identified hits, balanced pharmacodynamics (docking findings) and pharmacokinetics (ADME/Tox results) would need to be achieved via introducing ionizable scaffolds furnishing increased polarity while possessing relevant aromatic characteristics that are the advent for significant target affinity. Suggested scaffolds include tetrazole ring and other relevant cyclic carboxylate-related bioisosteres.

### 3.3. Molecular Dynamics Simulation Analysis

**Figure 3:**
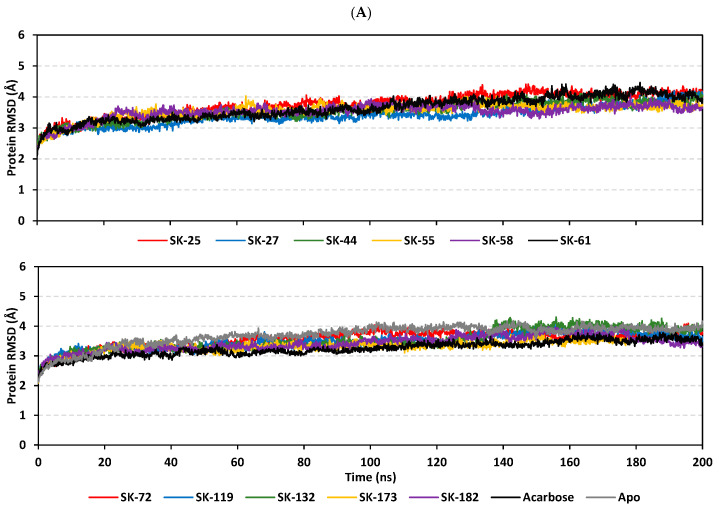

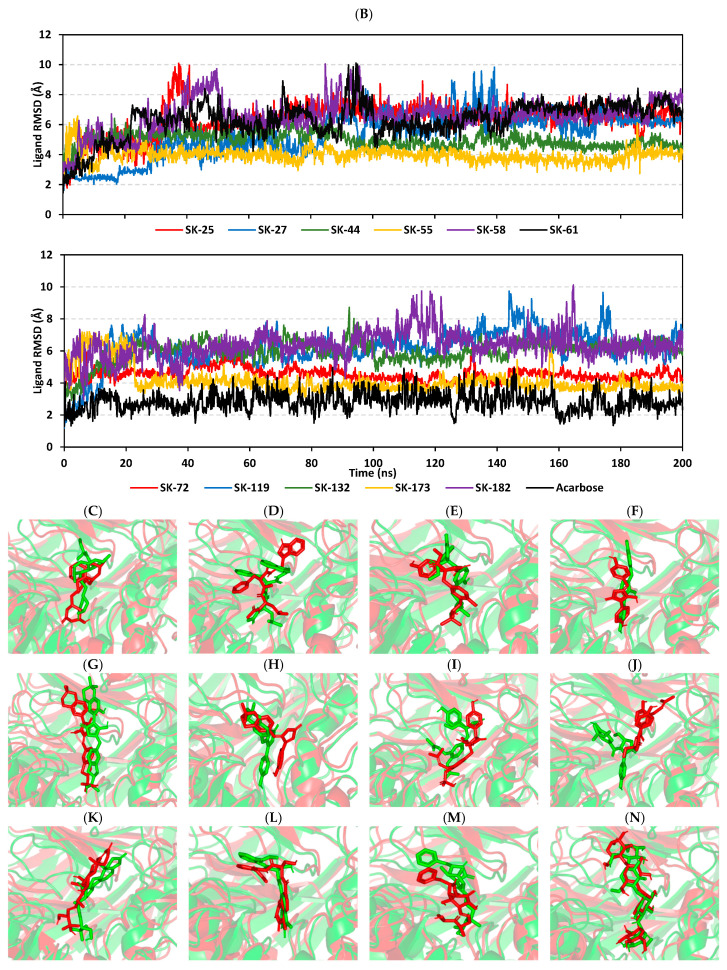
Thermodynamic stability of top-affinity *A. terrus*-isolated hits in the complex of the *hi*MGAM target. (**A**) Alpha-carbon RMSDs for target protein; (**B**) sole ligand’s RMSDs, in terms of simulation timeframes (ns). (**C**–**N**) Overlaid ligand-*hi*MGAM snapshots at initial and final timeframes; (**C**) SK-25, (**D**) SK-27, (**E**) SK-44, (**F**) SK-55, (**G**) SK-58, (**H**) SK-61, (**I**) SK-72, (**J**) SK-119, (**K**) SK-132, (**L**) SK-173, (**M**) SK-182, and (**N**) acarbose. Ligands (sticks) and bounded *hi*MGAM proteins (cartoons) are colored green and red, with respect to 0 ns and 200 ns extracted frames.

The thermodynamic behavior of *hi*MGAM complexes with identified hits was investigated in relation to the co-crystallized acarbose throughout explicit molecular dynamics simulations. Both hits with promising pharmacokinetic profiles and those of flagged parameters were enrolled within the molecular dynamics study. This would provide valuable insights relating to functional group-associated complex stability, the thing that would guide future structural modification and lead optimization. This would provide molecular insights in terms of target/ligand interactions under near-physiological conditions as well as validate the predicted ligand’s affinity, in a way, and being more sophisticated than flexible docking protocols [41,85,86]. In reference to corresponding initial structures, the root-mean standard deviation (RMSD) trajectories were monitored for each simulated protein and bounded ligand molecule; this allowed for investigating respective conformational/orientational alterations, as well as ensuring corresponding binding stability [87]. Generally, altered conformational profiles and compromised stabilities are correlated with high protein RMSD values; whereas, ligands with excellent pocket accommodation correspond to steady/small-valued ligand RMSD tones [88]. Typical thermodynamic behaviors were depicted for the simulated proteins since carbon-alpha RMSDs showed elevation across the initial times, owing to system relaxation, followed by leveled-off trajectories around respective averages for more than half of the simulation runs. Interestingly, monitored RMSDs for all ligand-bounded *hi*MGAM proteins were at relatively lower average values and less fluctuating trajectories as compared to the apo/unliganded protein (3.50 ± 0.13 Å versus 3.69 ± 0.35 Å) (Figure 3A). The latter apo versus holo dynamic behavior conferred the compactness and increase of stability for the complexed target proteins upon ligand binding. It is worth mentioning that all holo *hi*MGAM proteins managed to converge around a mean RMSD of 3.09 ± 0.12 Å for more than half of the simulation runs (~130 ns). The latter is adequate with relevant protein stability and sufficient convergence, as well as molecular dynamic validity, with no need for further time extensions.

Moving towards the sole ligand’s RMSDs, significant conferment/stability for the simulated ligands within the bound target binding site has been depicted (Figure 3B). Across the simulation runs, limited fluctuations and almost-steady trajectories were assigned for SK-44, SK-55, SK-72, and SK-173 (4.96 ± 0.47 Å, 3.98 ± 0.46 Å, 4.56 ± 0.39 Å, and 4.17 ± 0.81 Å) as compared to other simulated hits. Lower RMSD tones were assigned for SK-55 and SK-173, which conferred optimum ligand-pocket confinement and minimal conformational/orientation alterations for the simulated ligands. Concerning the other simulated hits, higher ligands’ RMSD tones and significant fluctuations were depicted across a few time frame ranges (~ 6.64 ± 1.10 Å). This could confer relevant orientation shifts at high RMSD trajectories, as well as compromised stability for the simulated ligand–target complexes. However, all of these less-stable models almost showed a steady-off RMSD around a comparable average value (6.16 ± 0.41 Å), beyond the 100 ns times and until the end of the simulation runs. Notably, the depicted RMSDs for all simulated hits never exceeded three-fold the values of their respective bound *hi*MGAM target proteins after reaching the respective simulation plateau (beyond 100 ns). This would confirm significant ligand existence within the target pocket and relevant complex stability, as well as successful protein convergence [89,90]. Regarding the co-crystallized ligand, acarbose’s RMSD trajectories were of the lowest average value (2.83 ± 0.96 Å); yet, they had higher fluctuations compared to top-stable hits (SK-44, SK-55, SK-72, and SK-173). The latter thermodynamic behavior would highlight the beneficial role of aromatic scaffolds incorporated within the top-stable hits. Providing significant hydrophobic π-mediated contacts could fortify the ligand’s stability over just Van der Waals forces of interactions via glycosidic hydrocarbons. However, these scaffolds should be at relevant positions on the ligand’s skeleton for achieving proper orientations and close-range contacts, as would be suggested for the top-stable hits. The ligand-pocket accommodation was further confirmed by monitoring the time evolution of ligand–target complex conformations and the ligand’s orientation over the overlaid timeframes at the beginning and end of the simulation runs (Figure 3C–N). Limited conformation/orientation changes were illustrated for the top-stable simulated hits (SK-44, SK-55, SK-72, and SK-173), as well as the co-crystallized acarbose at the end of the dynamic run; whereas, relevant alterations were noticed for the rest, particularly SK-27 and SK-119. This was consistent with the sole ligand’s RMSD tones.

**Figure 4:**
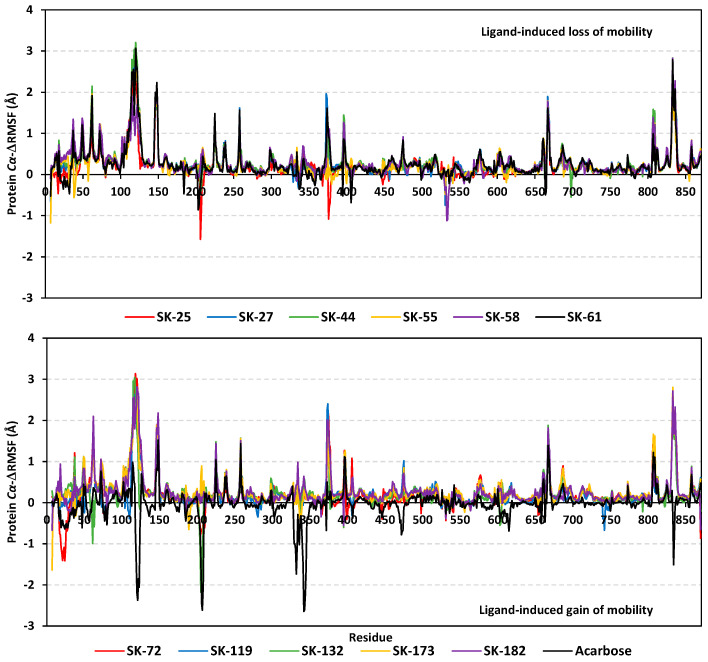
Analysis of *hi*MGAM ΔRMSF trajectories across the entirety of the molecular dynamics runs. Residue-wise flexibility contributions of the holo-target proteins are represented in relation to the apo/unliganded state. ΔRMSF trajectories are represented as per the amino acid sequence number (residues *N*-terminus Val7-to-His870 *C*-terminus).

Monitoring the RMS fluctuations (RMSFs) of the bound- (holo) and apo-target proteins in relation to their alpha-carbon references provided further stability analysis. The approach provided a dissection of the proteins flexibility/immobility characteristics, down to their constituting amino acids [91]. RMSFs would permit us to grasp the residue-wise dynamic behaviors at the protein’s binding pocket/vicinal loops, in addition to pinpointing the key amino acids for the ligand’s anchoring [92,93]. Difference root-mean-square fluctuation (ΔRMSF) was adopted as a better estimation of the protein’s local flexibility, which comprises the RMSF difference for each *hi*MGAM holo protein in relation to the apo state (ΔRMSF = apoRMSF—holoRMSF). Adopting a ΔRMSF cut-off value of 0.30 Å was relevant for estimating the significant alterations within the protein’s structural movements, meaning that residues depicting ΔRMSF beyond 0.30 Å illustrated limited mobility [94]. Typical dynamic behavior was depicted since there were higher immobility profiles for the far amino terminal residues as compared to core ones, as well as for the carboxy-terminal residues as compared to *N*-terminus (Figure 4). This was in good agreement with the molecular dynamics nature of the isolated *hi*MGAM proteins isolated by Zhang et al. [95], in addition to the B-factor analyses and conformational stability findings reported for several crystallized *hi*MGAM structures [60,61,62,63,65]. These findings could ensure the validity of the conducted molecular dynamics simulations, as well as the adopted protocol.

As a general observation, trends of more positive or less negative ΔRMSFs were depicted for the residue-bound top-stable hits (SK-44, SK-55, SK-72, and SK-173) as compared to other simulated hits or even the reference acarbose. This was most obvious across the residue range Thr204-to-Gly208, showing ΔRMSFs reaching down to –1.57 Å, –2.18 Å, and –2.61 Å for SK-25, SK-132 and acarbose, respectively. Other high-flexibility regions include Gly533-to-Phe535, Ser376-to-Pro378, and Phe119-to-Ala123/Asp340-to-Lys345, being solely depicted for SK-58, SK-25, and acarbose, respectively, inferring the negligible contribution of such residues for ligand–protein stability. Several mobility regions being assigned to the simulated acarbose model would further highlight the impact of acarbose’s fluctuations on the stability of the protein’s tertiary structure, the thing that could be correlated to an increased number of acarbose’s incorporated rotatable bonds as compared to all simulated hits. It is worth noting that, most protein regions are within the positive ΔRMSF range, the thing that confirmed the gained stability of the *hi*MGAM proteins following the binding of hit compounds. These insights were in agreement with the above-furnished RMSD trajectory analysis.

The comparable preferential stability patterns above were illustrated down to the dissected residue-wise fluctuations within each *hi*MGAM key binding domain and the secondary structure/functional motifs (Table 3). The residues of the substrate’s binding site, including Arg298, Tyr299, Asp327, Ile328, Phe575, Ala576, Leu577, His600, Gln603, and/or Phe605, predicted recognized immobility profiles (ΔRMSF up to 0.67 Å), the thing that highlights their crucial role in ligand anchoring at the –1 sugar subsite. Wider residue-wise stability ranges were assigned for SK-44, SK-44, SK-72, and SK-173 as compared to other ligands. Regarding domain preferentiality, residue-wise rigidity/stability was in favor of the catalytic GH-31 domain residues, as well as both inserted loops. Limited residues of the *N*-terminal *β*-domain were suggested for a relevant role in stabilizing the simulated ligands at the *hi*MGAM substrate-binding site. Based on the hydrophobic/hydrophilic nature of the depicted immobilized residues, trends of polar residues were dominant for stabilizing the acarbose–protein model as both polar and hydrophobic amino acids were shown to be important for hit stability. This further highlights the beneficial role of the incorporated aromatic scaffold for the hit’s pharmacodynamic preferentiality and affinity towards the target.

**Figure 5:**
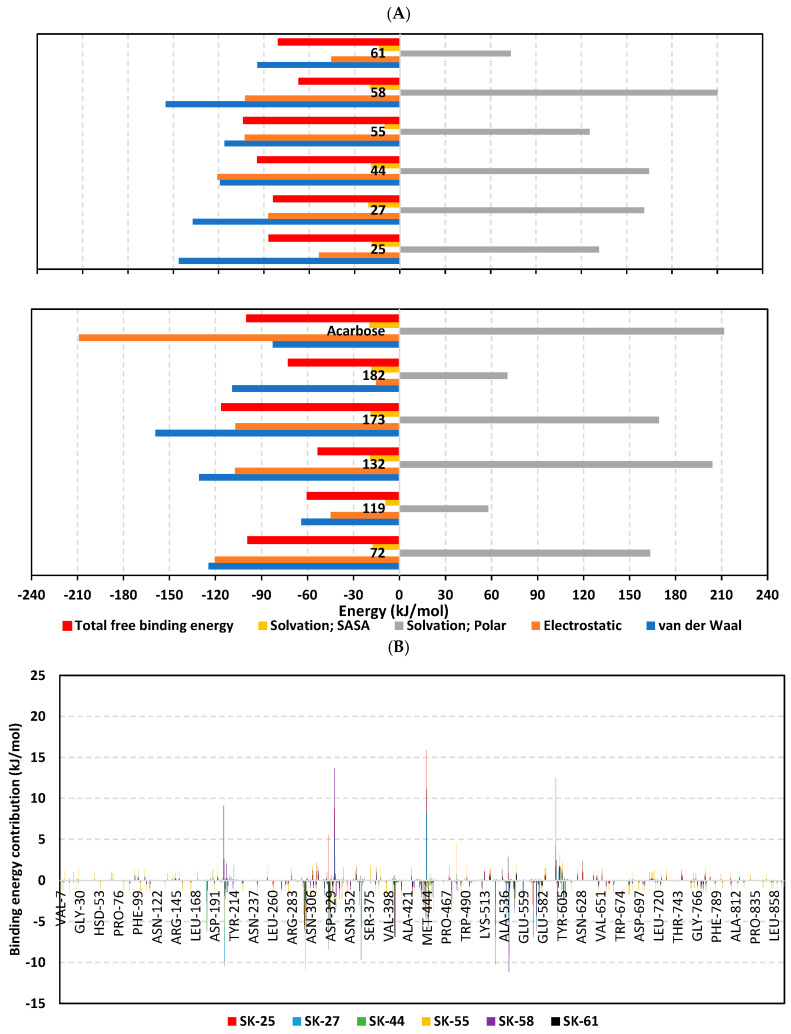

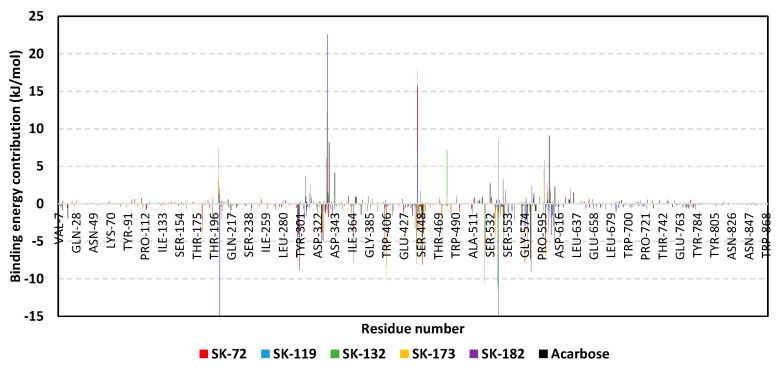
MM_PBSA free binding energy calculations for ligand-*hi*MGAM complexes. (**A**) Total free binding energies and their constituting energy terms. (**B**) Residue-based energy contributions.

The free binding energy for hit–protein complexes was estimated using the trajectory-oriented molecular mechanics_Poisson–Boltzmann surface area (MM_PBSA) approach in order to understand the binding nature, estimate affinity magnitudes, and pinpoint the individual energy contribution of key pocket residues [96]. Typically, MM_PBSA is reported with comparable accuracy to free-energy perturbations; however, this is achieved with much lower computational expenders [51]. A single trajectory approach and SASA-only model were adopted for free binding energy calculations, where higher negative binding energy explains the preferential ligand’s affinity. Notably, the total binding energy of the simulated SK-55 and SK-173 came to be higher than the acarbose reference *hi*MGAM inhibitor exhibiting superior binding affinities (–103.46 ± 14.56 kJ/mol and –116.05 ± 21.13 kJ/mol versus –99.60 ± 22.81 kJ/mol), as illustrated within Figure 5A. Relatively lower negative free binding energies were deduced for SK-44 and SK-72 (–94.12 ± 25.83 kJ/mol and –95.88 ± 21.74 kJ/mol, respectively) as much lower values were assigned for other simulated hits (from –53.03 ± 16.66 kJ/mol and up to –86.69 ± 31.43 kJ/mol). The latter preferential affinity patterns were in good translation for the previously described RMSD and RMSF analysis, as well as preliminary docking results.

Dissecting the obtained binding-free energy and its contributing energy terms showed a dominant energy contribution of the electrostatic interactions for acarbose reference. The superior electrostatic potential is beyond five-fold that of the hydrophobic Van der Waals contribution forces. This could be the reason for the wide range of polar oxygen-related functionalities incorporated within acarbose structure in relation to its hydrocarbon skeleton. Findings regarding electrostatic preferentiality were also reported by Mahmud et al. through their investigation of natural phenolics as potential alpha-glucosidase inhibitors [97]. Similar findings were seen through the molecular dynamic simulation of apigenin-7-*O*-glucoside with an alpha-glucosidase target [98]. On the other hand, the top-stable hits (SK-44, SK-55, and SK-72) showed almost-balanced electrostatic/hydrophobic contributions since the energy terms were nearly comparable. This was suggested to be related to their comparable hydrogen bonding group-to-aromatic ratios. This was consistent with reported phthalimide-benzenesulfonamides, where these hybrids showed great affinity towards alpha-glucosidase targets owing to their balanced hydrophobic/polar profiles [99]. Notably, the ionizable top-stable hit, SK-173, showed preferential electrostatic interaction compared to the hydrophobic Van der Waals forces, the thing that could be related to its ionizable sulphonic acid group. On the contrary, the rest of the simulated hits depicted superior Van der Waals forces over electrostatic interactions owing to their dominant non-polar functionalities.

Regarding the penalty energy contributions, polar solvation energies reached their highest values with acarbose at 211.60 ± 29.71 kJ/mol, followed by several hits, including SK-132 and SK-58 (204.01 ± 7.32 kJ/mol and 210.23 ± 3.99 kJ/mol, respectively). Positive solvation energy contribution is generally detrimental to ligand–protein binding since binding is a solvent-displacement process [100]. Increased polar solvation energy for acarbose could be the reason for oxygen functionalities. Despite being important for furnishing polar contact with -1 and +1 sugar subsites, they can act as double blades increasing solvation entropy since the target pocket is shallow and solvent-exposed [7,36]. On the other hand, increased ligand hydrophobicity for SK-132 and SK-58, particularly through aromatic scaffolds, could rationalize a significant solvation penalty. Reported evidence regarding the hydration network and accumulation of highly ordered water molecules at the ligand’s hydrophobic surfaces were reported as significant for hampering ligand–target binding and increasing solvation entropy [100,101]. It is worth mentioning that the top-stable ligands showed relevant solvation penalty, with values ranging from 125.43 ± 22.07 kJ/mol for SK-55 and around 165.00 kJ/mol for SK-44, SK-72 and SK-173. Despite illustrating such relevant penalties, depicting high contributions of hydrophobic and electrostatic potentiality allowed reasonable overcompensation of solvation entropy and final high total free binding energy profiles. Such findings further highlight the structural postulation presented at docking and the ADME/Tox investigation, where providing balanced hydrophobic/polar structural features, as with the ionizable tetrazole ring with aromatic/lipophilic features, would be able to minimize solvation penalty and maximize binding affinities.

For gaining more insights concerning ligand–residue interactions, the binding-free energy decomposition was applied to identify the key residues involved in the obtained total binding-free energies [51]. Residues of the active binding site showed favored contributions (high negative values) within the ligand–protein binding energies of almost all ligands (Figure 5B). Pocket residues, such as Asp203, Arg298, Tyr299, Glu300, Asp327, Asp329, catalytic Glu404, Asp443, Glu446, Arg526 catalytic Asp542, Asp571, and/or His600, were depicted as important for complex stability showing high negative binding contributions (≥ −3.00 kJ/mol). Several of these polar residues were of higher negative values in systems of the top-docked hits, as well as the acarbose reference ligand. The depicted ligand–residue pattern was in good agreement with reported molecular dynamics studies for both nature-isolated and synthesized compounds [36,97,98,99,102]. On the other hand, the energy contributions of several hydrophobic pocket residues, such as Trp299, Ile328, Ile364, Val405, Trp406, Trp441, Val447, Trp539, and Phe575, were only significant for the aromatic/hydrophobic hits (up to −10.27 kJ/mol for Trp406). Notably, fewer residues, such as Arg202, Met444, Arg526, and Arg598, showed significant positive energy contributions, inferring the repulsion forces and unfavored impact on the ligand’s stability. It is worth mentioning that these energy residue-wise findings were consistent with the above-described ΔRMSF hydrophobic/polar contact preferentiality.

## 4. Conclusions

The outcomes of our study highlight the promising molecular interactions and affinities of the marine-derived *A. terreus* isolates towards the human α-glucosidase target, withholding potentiality as compared to acarbose, the market drug reference. Combined molecular docking and dynamics simulations ensured the effective binding of the eleven top-affinity hits, with predominant stabilities being assigned for butyrolactone VI (SK-44), aspulvinone E (SK-55), butyrolactone I 4′′′′-sulfate (SK-72), and terrelumamide B (SK-173). The top-promising molecules harbor a relevant polar hydrogen bond warhead, which was relevant for deep insertion within the –1 carbohydrate subpocket and mediating interactions with the catalytic residues. Additionally, having hydrophobic extended tail groups has been advantageous for those outwards reaching towards the +2 and/or +3 subpockets, permitting extra stability. Relevant drug-likeness profiles and low toxicity potentiality have been presented for most investigated hits through ligand-based in silico pharmacokinetic ADME/Tox studies. Insights of increasing the compound’s polarity and lowering the hydrophobic characteristics highlight a better gut brush border concentration and minimal toxicity profiles for ligand hits SK-72, terrelumamide B (SK-173), and SK-182. Thermodynamic stability highlighted the importance of the high energy contributions of hydrophobic and electrostatic potentials for permitting the reasonable overcompensation of solvation entropy, as well as furnishing final high total free binding energy profiles. In consistency with docking and ADME/Tox studies, structural modifications for balanced hydrophobic/polar structural features, as with an ionizable tetrazole ring or other carboxylate bioisosteres, would be able to minimize the solvation penalty and maximize binding affinities. Prospective work concerning enhanced sampling simulation, in vitro experimental studies, and animal model testing is required for optimizing and developing clinical candidates based on the entitled *A. terreus*-derived promising hits.

## Figures and Tables

**Table 1:** Docking affinities and characteristics for ligand–target interactions at *hi*MGAM’s substrate pocket across high-precision molecular docking protocol.

Compound	Affinity Energy (Kcal/mol)	H-bond Interactions [Length (Å); Angle (°); Binding Residues]	Hydrophobic Interactions	π-Driven Interactions
**Acarbose** Co-crystalline Ligand **ACA** 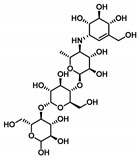	–11.388	1.90; 158; Asp203 (sidechain C**O**^−^/6-deoxyglucosyl 3′-O**H**) 2.00; 159; Asp203 (sidechain C**O**^−^/6-deoxyglucosyl 4′-O**H**) 2.10; 163; Thr205 (sidechain **O**H/+3 maltosyl 6′-O**H**) 1.90; 171; Asp327 (sidechain C**O**^−^/valienamine 4′-O**H**) 2.00; 165; Arg526 (sidechain = NH**H**/6-deoxyglucosyl 3′-**O**H) 2.00; 145; Arg526 (sidechain = N**H**H/valienamine 6′−**O**H) 1.90; 143; Asp542 (sidechain C**O**^−^/glycosidic linker N**H**) 1.70; 157; Asp542 (sidechain C=**O**/valienamine 6′−O**H**) 2.30; 145; His600 (sidechain N**H**/valienamine 4′−**O**H) 2.30; 137; His600 (sidechain N**H**/valienamine 5′−**O**H)	Tyr299, Ile328, Ile364, Trp406, Trp441, Phe450, Trp539, Phe575, Ala576, Leu577, Tyr605	—
**Lovastatin** CID: 53232 **SK-25** 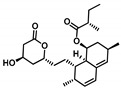	–9.071	2.10; 157; Asp327 (sidechain **O**^−^/4-O**H**) 3.10; 135; Asp327 (sidechain C=**O/**4-O**H**) 2.00; 132; Arg526 (sidechain N^+^H**H**/lactone C=**O**)	Tyr299, Ile328, Ile364, Trp406, Trp441, Met444, Phe450, Trp539, Phe575, Ala576, Leu577, Tyr605	—
**Aspergillamide A** CID: 6917355 **SK-27** 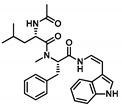	–8.747	2.30; 132; Arg526 (sidechain N^+^H**H**/peptide C=**O**) 2.10; 125; Asp542 (sidechain C=**O**/peptide N**H**) 2.00; 167; His600 (sidechain N**H**/peptide C=**O**)	Tyr299, Ile328, Ile364, Trp406, Trp441, Met444, Phe450, Trp539, Phe575, Ala576, Leu577, Tyr605	Phe575
**Butyrolactone VI** CID: 46930025 **SK-44** 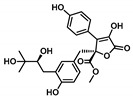	–11.206	2.50; 171; Asp203 (sidechain **O**^−^/phenolic O**H**) 3.40; 129; Trp406 (sidechain N**H**/tail **O**H) 2.00; 169; Asp443 (sidechain **O**^−^/tail O**H**) 2.10; 160; Arg526 (sidechain N^+^H**H**/phenolic O**H**)	Tyr299, Ile328, Ile364, Trp406, Trp441, Met444, Phe450, Trp539, Phe575, Ala576, Leu577, Tyr605	Tyr299 Trp406
**Aspulvinone E** CID: 54675753 **SK-55** 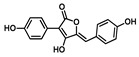	–11.789	2.10; 138; Asp327 (sidechain **O**^−^/phenolic O**H**) 2.40; 160; Asp327 (sidechain C=**O**/phenolic O**H**) 2.30; 167; Arg526 (sidechain N^+^H**H**/furan **O**H) 3.50; 132; Asp542 (sidechain **O**^−^/furan O**H**) 3.00; 139; His600 (sidechain N**H**/phenolic **O**H)	Tyr299, Ile328, Ile364, Trp406, Trp441, Met444, Phe450, Trp539, Phe575, Ala576, Leu577, Tyr605	Tyr299 Trp406 Phe450 Phe575
**Aspulvinone F** CID: 54728278 **SK-58** 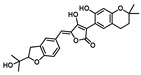	–10.065	2.10; 167; Asp327 (sidechain **O**^−^/2-propan O**H**) 3.10; 132; Asp327 (sidechain C=**O**/2-propan O**H**) 3.10; 139; Thr205 (sidechain **O**CH_3_/furan C=**O**)	Tyr299, Ile328, Ile364, Trp406, Trp441, Met444, Phe450, Leu473, Trp539, Phe575, Ala576, Leu577, Tyr605	Trp406
**Rubrolide S** CID: 101885283 **SK-61** 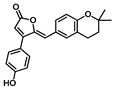	–9.469	3.50; 124; Asp203 (sidechain **O**^−^/tautomeric furan C=**O**) 2.10; 145; Asp327 (sidechain **O**^−^/phenolic O**H**)	Tyr299, Ile328, Ile364, Trp406, Trp441, Met444, Phe450, Trp539, Phe575, Ala576, Leu577, Tyr605	Trp299 Phe575 Phe575
**Butyrolactone I** **4′′′′-Sulfate** CID: 91935887 **SK-72** 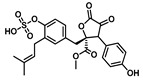	–11.039	2.00; 173; Thr205 (sidechain **O**CH_3_/phenolic O**H**) 3.40; 168; Trp406 (sidechain N**H**/S-**O**^−^) 2.60; 129; Arg526 (sidechain N^+^H**H**/S-**O**H) 2.00; 126; Asp542 (sidechain **O**^−^/S-O**H**) 2.50; 170; Asp542 (sidechain C=**O**/S-O**H**) 3.30; 168; His600 (sidechain N**H**/S=**O**)	Tyr299, Ile328, Ile364, Trp406, Trp441, Met444, Phe450, Trp539, Phe575, Ala576, Leu577, Tyr605	Trp406
(**+**)−**Asperteretone F** CID: 156582453 **SK-119** 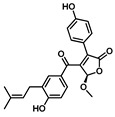	–9.847	2.60; 157; Asp327 (sidechain **O**^−^/phenolic O**H**) 2.60; 171; Arg526 (sidechain N^+^H**H**/phenolic **O**H) 3.20; 146; His600 (sidechain N**H**/furan C=**O**)	Tyr299, Ile328, Ile364, Trp406, Trp441, Met444, Phe450, Trp539, Phe575, Ala576, Leu577, Tyr605	Tyr299 Phe575
**12,15,25,28-tetrahydroxyergosta-4,6,8**(**14**)**,22-tetraen-3-one** **SK-132** 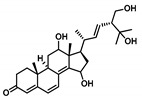	–10.184	2.10; 159; Asp203 (sidechain **O**^−^/C15 βO**H**) 2.90; 124; Asp203 (sidechain C=**O**/C15 βO**H**) 2.40; 122; Asp327 (sidechain C=**O**/C25 O**H**) 1.90; 159; Asp443 (sidechain **O**^−^/C26 O**H**)	Tyr214, Tyr299, Ile328, Ile364, Trp406, Trp441, Met444, Phe450, Val451, Trp539, Phe575, Ala576, Leu577, Tyr605	—
**Terrelumamide B** CID: 139586668 **SK-173** 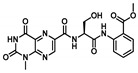	–11.565	2.70; 141; Asp203 (sidechain **O**^−^/CH_2_O**H**) 2.30; 121; Asp203 (sidechain C=**O**/CH_2_O**H**) 2.20; 158; Thr205 (sidechain O**H**/benzamide C=**O**) 3.10; 126; Asp327 (sidechain C**OO**^−^/tautomeric 2-C=**O**/Enol) 1.90; 128; Arg526 (sidechain N^+^H**H**/tautomeric 4-C=**O**/Enol) 2.70; 126; Asp542 (sidechain C**OO**^−^/tautomeric 2-C=**O**/Enol) 1.90; 156; Asp542 (sidechain **O**^−^/carboxamide linker N**H**) 3.30; 138; His600 (sidechain N**H**/tautomeric 2-C=**O**/Enol)	Pro206, Tyr214, Tyr299, Ile328, Ile364, Trp406, Trp441, Met444, Phe450, Trp539, Phe575, Ala576, Leu577, Tyr605	Tyr299 Trp406 Phe575
**Cytochalasin Z11** CID: 24970396 **SK-182** 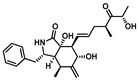	–10.278	2.30; 133; Asp327 (sidechain **O**^−^/tail O**H**) 3.30; 123; Arg526 (sidechain N^+^H**H**/tail **O**H) 2.70; 127; Asp547 (sidechain **O**^−^/ ring junction αO**H**) 1.90; 153; His600 (sidechain N**H**/tail **O**H)	Pro206, Tyr299, Ile328, Ile364, Trp406, Trp441, Met444, Phe450, Trp539, Phe575, Ala576, Leu577, Tyr605	Phe450

**Table 2:** In silico pharmacokinetic and safety (ADME/TOX) ^a^ characteristics for screening hits and co-crystallized ligands.

Comp.	“RO’5” HBD; HBA; θ; M.Wt.	PlogP −2.0 to 6.5	PlogS mol/dm^3^ −6.5 to 0.5	PPCaco nm/sec <25 Poor >500 Great	%HOA <25% Poor >80% Great	PPMDCK nm/sec <25 Poor >500 Great	PlogBB −3.0 to 1.2	PlogK_HSA_ −1.5 to 1.5	PlogHERG Significant Block > –5.0	Oral Rat LD_50_ mg/Kg	AMES Mutagenesis (Predicted Index)
**SK-25**	1; 5; 7; 404.55	4.34	−4.57 (Moderate solubility)	717.35	95%	345.47	−1.03	0.71	–4.78	556.97	Negative (0.07)
**SK-27**	3; 7; 10; 474.61	3.33	–4.91 (Moderate solubility)	1146.17	100%	792.72	–0.79	0.04	–2.47	779.48	Negative (0.18)
**SK-44**	5; 9; 8; 401.17	2.43	–3.70 (Soluble)	356.17	82%	804.20	–0.81	0.65	–4.32	203.09	Negative (0.48
**SK-55**	3; 5; 2; 296.28	2.60	–3.61 (Soluble)	363.07	83%	921.33	–0.77	0.54	–4.98	110.64	Negative (0.32)
**SK-58**	3; 7; 3; 464.51	4.81	–5.28 (Moderate solubility)	893.74	89%	633.46	−0.88	0.79	–6.70	334.28	Negative (0.32)
**SK-61**	1; 4; 2; 348.40	4.72	–4.82 (Moderate solubility)	623.18	91%	296.72	–0.89	0.78	–5.98	320.74	Negative (0.25)
**SK-72**	2; 10; 9; 504.51	–0.29	–5.00 (Moderate solubility)	5.51	39%	2.28	–2.75	–0.40	–3.52	1520.26	Negative (0.22)
**SK-119**	2; 6; 6; 394.42	4.23	–4.79 (Moderate solubility)	229.29	83%	100.69	–1.48	0.02	–5.16	486.10	Negative (0.04)
**SK-132**	4; 5; 5; 456.62	2.27	–3.31 (Soluble)	123.45	84%	51.57	–1.91	0.50	–4.62	1900.59	Negative (0.07)
**SK-173**	4; 13; 7; 442.39	–0.82	–1.99 (High solubility)	16.21	40%	5.75	–3.08	–0.46	–6.09	2008.27	Negative (0.35)
**SK-182**	4; 6; 7; 427.54	1.74	–3.29 (Soluble)	73.14	73%	62.69	–1.98	–0.14	–4.15	1033.63	Negative (0.26)
**Acarbose**	14; 19; 9; 645.61	–5.51	–2.13 (Extreme solubility)	0.05	0%	0.01	–5.57	–2.54	–5.62	24,405.50 23,989.66 *	Negative (0.03)

^a^ RO’5; hydrogen-bond donors (HBDs) below or equal to 5, hydrogen bond acceptors (HBAs) below or equal to 10; rotatable bonds (θ) below 5, M.Wt below 500 g/mol, logP_o/w_ < 5. PlogP_o/w_ partition coefficient at system octanol/water; PlogS aqueous solubility; PPCaco permeation across Caco2-cells modeling the gut–blood barrier; PlogBB blood–brain partition coefficient modeling blood–brain barrier”; PPMDCK permeation across Madin–Darby’s dog kidney cells modeling the blood–brain barrier; PlogK_HSA_ human serum albumin conjugation; % HOA human oral absorption in percentages; PlogHERG half-maximal inhibition concentration for the human ether-a-go-go-related gene (HERG)_K_v_11.1-channel blockage. Recommended and/or accepted values via QikProp software. * Deposited experimental data within TEST software.

**Table 3:** Difference RMSF ^a^ for bounded *hi*MGAM proteins across the 200 ns molecular dynamics simulations.

Canonical Domains Comprising Substrate Pocket ^b^	Residues	SK-25	SK-27	SK-44	SK-55	SK-58	SK-61	SK-72	SK-119	SK-132	SK-173	SK-182	Acarbose
***N*-terminus *β*-sheet domain**	**Arg202**	0.01	0.15	0.03	0.12	–0.11	0.00	–0.38	0.09	–0.06	–0.09	0.07	–0.34
	**Asp203**	−0.15	0.25	0.20	0.22	–0.03	–0.86	–0.38	0.14	–0.52	0.20	0.21	–0.40
	**Thr204**	−0.06	0.19	0.17	0.19	–0.07	–0.76	–0.60	0.09	–1.18	0.07	–0.01	–0.76
	**Thr205**	–0.02	0.12	0.01	0.22	0.10	–0.38	–0.68	0.11	–2.18	**0.64**	0.13	–1.42
	**Pro206**	–1.57	0.02	–0.34	0.27	0.23	–0.29	–0.65	0.17	–2.10	**0.89**	0.03	–2.47
	**Asn207**	–0.98	**0.30**	–0.39	**0.41**	**0.50**	–0.08	–0.78	**0.35**	–1.16	0.08	0.13	–2.61
	**Asn209**	–0.14	0.29	0.15	**0.66**	**0.61**	**0.44**	–0.15	**0.32**	0.25	**0.53**	0.27	–0.58
	**Thr211**	0.02	0.25	0.12	0.43	0.20	0.17	–0.20	0.00	0.36	0.24	–0.10	0.15
	**Tyr214**	0.07	0.21	0.16	0.19	0.07	0.17	0.06	0.14	0.09	0.14	0.18	0.09
**GH-31 catalytic domain**	**Arg298**	**0.31**	**0.52**	**0.52**	**0.57**	**0.66**	**0.59**	**0.47**	**0.56**	**0.59**	**0.46**	0.27	**0.30**
	**Tyr299**	**0.52**	**0.45**	**0.49**	**0.55**	**0.59**	**0.58**	**0.44**	**0.43**	**0.56**	**0.33**	–0.02	0.01
	**Asp327**	**0.38**	0.13	**0.38**	**0.46**	**0.48**	0.26	**0.34**	**0.35**	**0.34**	**0.33**	0.28	**0.40**
	**Ile328**	0.26	0.09	0.29	**0.43**	**0.39**	0.20	0.10	**0.31**	0.26	**0.49**	0.20	–0.24
	**Ile364**	0.08	0.03	0.09	0.17	0.15	0.07	0.06	0.06	0.08	0.07	0.03	–0.02
	**Trp441**	0.04	0.02	0.05	0.05	0.01	0.04	–0.01	–0.04	–0.03	0.03	0.03	–0.07
	**Asp443**	0.08	0.11	0.10	0.06	0.08	0.12	0.02	0.12	0.00	0.15	0.10	–0.25
	**Met444**	0.06	0.12	0.09	0.14	0.10	0.10	–0.20	0.11	0.07	0.16	0.14	–0.19
	**Ser448**	–0.24	0.08	0.09	0.05	0.08	0.01	–0.13	0.14	0.05	0.07	0.13	–0.18
	**Arg526**	**0.31**	0.07	–0.11	0.07	–0.05	–0.10	–0.15	–0.11	–0.05	–0.04	0.04	**0.31**
	**Trp539**	0.15	–0.05	0.02	0.11	0.09	0.09	–0.13	0.04	0.10	0.08	0.27	0.10
	**Gly541**	0.09	–0.21	–0.02	–0.22	0.05	0.10	–0.24	0.09	0.06	0.16	–0.12	–1.04
	**Asp542**	**0.42**	0.03	0.19	0.09	0.06	0.15	0.10	0.02	0.02	0.10	0.08	**0.43**
	**Asp571**	0.08	0.13	0.16	0.20	0.16	0.17	0.19	0.11	0.13	0.17	0.18	0.01
	**Phe575**	**0.32**	0.15	**0.48**	0.16	**0.36**	**0.42**	**0.56**	0.29	**0.30**	**0.54**	**0.31**	0.14
	**Ala576**	**0.46**	**0.47**	**0.50**	**0.33**	**0.55**	**0.50**	**0.62**	**0.48**	**0.42**	**0.47**	**0.35**	0.11
	**Leu577**	**0.44**	**0.61**	**0.53**	**0.42**	**0.55**	**0.61**	**0.67**	**0.55**	**0.42**	**0.55**	**0.45**	0.16
	**Arg598**	0.01	0.03	0.03	–0.04	0.10	0.04	0.02	0.06	0.00	0.04	0.06	–0.04
	**His600**	**0.33**	0.12	**0.45**	**0.51**	0.21	0.23	0.12	0.13	0.13	0.21	0.19	**0.33**
	**Gly602**	**0.45**	**0.40**	**0.40**	**0.43**	**0.37**	0.22	**0.36**	0.03	–0.09	**0.46**	0.27	–0.10
	**Gln603**	**0.56**	**0.54**	**0.38**	**0.64**	0.19	**0.55**	**0.48**	0.27	–0.55	**0.30**	**0.33**	–0.27
	**Phe605**	**0.40**	**0.50**	**0.44**	**0.45**	**0.39**	**0.44**	**0.43**	0.23	–0.37	**0.55**	0.27	–0.27
**Insert-I catalytic loop**	**Val405**	0.08	–0.02	0.05	0.26	0.09	–0.09	**0.42**	–0.01	0.22	**0.30**	0.20	–0.07
	**Trp406**	**0.32**	0.06	0.14	**0.39**	0.11	–0.68	**1.08**	–0.31	**0.49**	**0.44**	**0.57**	–0.10
**Insert-II catalytic loop**	**Ser448**	–0.24	0.08	**0.39**	**0.35**	0.08	0.01	**0.33**	0.14	0.05	0.07	0.13	–0.18
	**Phe450**	–0.11	0.01	**0.37**	**0.31**	0.00	0.08	0.25	0.14	0.16	0.10	0.20	–0.11
	**Leu473**	0.21	0.27	0.18	**0.34**	**0.44**	**0.47**	**0.37**	**0.50**	0.12	**0.49**	0.28	–0.68
	**Asp474**	**0.57**	**0.54**	**0.37**	**0.62**	**0.59**	**0.70**	**0.41**	**0.80**	0.20	**0.51**	**0.65**	–0.68

^a^ Estimated difference RMS fluctuations (ΔRMSFs) were determined for each hit-bound *hi*MGAM protein in relation to the apo/unliganded protein form. Residues showing significant immobility with a ΔRMSF ≥0.30 Å cut-off are being shown in bold red text. ^b^ Domain sections are color coded as per their locations within the target structure.

## Data Availability

Data are available in the manuscript.

## Supplementary Materials

The following supporting information can be downloaded at: https://www.mdpi.com/article/10.3390/metabo13080942/s1, Table S1 Designated chemical library of *A. terrus*-isolated metabolites.
