# Supplementary material for: Deciphering Molecular Aspects of Potential α-Glucosidase Inhibitors within Aspergillus terreus: A Computational Odyssey of Molecular Docking-Coupled Dynamics Simulations and Pharmacokinetic Profiling
Source: Metabolites. 2023 Aug 12;13(8):942. doi: 10.3390/metabo13080942 (PMC10456934; doi:10.3390/metabo13080942)
Supplement: Supplementary file 1 [file metabolites-13-00942-s001.zip › metabolites-2510088-supplementary.pdf]

metabolites

MDPI

Deciphering Molecular Aspects of Potential *α*-Glucosidase Inhibitors within *Aspergillus terreus*: A Computational Odyssey

Supporting data

Table S1. Designated chemical library of *Aspergillus terreus*-isolated metabolites

Compound name; Isomeric SMILES	Ligand Code	MW	TPSA
**2*R*-(-)-6-hydroxymellein**<br>O=C1C2=C(C=C(O)C=C2O)C[C@@H](C)O1	SK–001	194.19	66.76
**(-)-(3*R*)-6-methoxymellein**<br>O=C1C2=C(C=C(OC)C=C2O)C[C@@H](C)O1	SK–002	208.21	55.77
**6-(4'-hydroxy-2'-methyl phenoxy)-(-)-(3*R*)-mellein**<br>O=C1C2=C(C=C(OC3=CC=C(O)C=C3C)C=C2O)C[C@@H](C)O1	SK–003	300.31	76.00
**(3*R*,4*R*)-6,7-dimethoxy-4-hydroxymellein**<br>O=C1C2=C(C=C(OC)C(OC)=C2O)[C@@H](O)[C@@H](C)O1	SK–004	254.24	85.23
**Asperterrestide B**<br>O=C([C@@H]([C@@H](O)C1=CC=CC=C1)N(C)C([C@@H](C(C)C)NC([C@@H]<br>(C)N2)=O)=O)NC3=CC=CC=C3C2=O	SK–005	466.54	127.83
**Asperterrestide A**<br>O=C([C@@H]([C@@H](O)C1=CC=CC=C1)N(C)C([C@@H](C(CC)C)NC([C@@H]<br>(C)N2)=O)=O)NC3=CC=CC=C3C2=O	SK–006	480.56	127.83
**Arisugasin H**<br>CC(O[C@@H]1C[C@H](O)[C@]2(C)[C@@]3(O)CC4=C(C=C(C5=CC=C(OC)C=C5)O<br>C4=O)O[C@]3(C)CC[C@@]2(O)C1(C)C)=O	SK–007	526.60	135.67
**Arisugasin D**<br>CC(O[C@@H]1CC[C@]2(C)[C@@]3(O)CC4=C(C=C(C5=CC=C(OC)C=C5)OC4=O)O<br>[C@]3(C)CC[C@@]2(O)C1(C)C)=O	SK–008	512.60	115.44
**Terretrione B**<br>O=C(C(C(C)C)N(C)C(C1CC2=CC=CC=C2)=O)N(C)C1=O	SK–009	302.37	57.69
**Terretrione C**<br>O=C(C(C(CC)C)N(C)C(C1CC2=CC=CC=C2)=O)N(C)C1=O	SK–010	316.40	57.69
**1-methyl emodin**<br>O=C1C2=C(C=C(C)C=C2OC)C(C3=CC(O)=CC(O)=C13)=O	SK–011	284.27	83.83
**Aspernolide A**<br>O=C([C@](C(C1=CC=C(O)C=C1)=C2O)(CC3=CC(CCC(C)(C)O4)=C4C=C3)OC2=O)OC	SK–012	424.45	102.30
**Aspernolide F**<br>O=C([C@](C(C1=CC=C(O)C=C1)=C2OCC)(CC3=CC=C(O)C(C/C=C(C)\C)=C3)OC2=O) OC	SK–013	452.50	102.30
**Aspernolide B**<br>CC(C)(CCC1=C(C=CC(=C1)C[C@@]2(C(=C(C(=O)O2)O)C3=CC=C(C=C3)O)C(=O)OC) O)O	SK–014	442.46	133.52
**Aspernolide C**<br>CC(C)(CCC1=C(C=CC(=C1)C[C@@]2(C(=C(C(=O)O2)O)C3=CC=C(C=C3)O)C(=O)OC) O)OC	SK–015	456.49	122.53
**Aspernolide E**<br>O=C([C@](C(C1=CC=C(O)C=C1)=C2O)(CC3=CC(C=CC(C)(C)O4)=C4C=C3)OC2=O)OC	SK–016	422.43	102.30
**Aspernolide G**<br>O=C([C@](C(C1=CC=C(O)C=C1)=C2OCC)(CC3=CC=C(O)C(C[C@H](O)C(C)(O)C)=C3)<br>OC2=O)OC	SK–017	486.52	142.76
**1,8-dihydroxy-3-methoxy-6-methylanthracene-9,10-dione**<br>O=C1C2=C(C=C(C)C=C2O)C(C3=CC(OC)=CC(O)=C13)=O	SK–018	284.27	83.83
**Methyl, 6-acetyl-4-methoxy-5,8-dihydroxynaphthalene-2-carboxylate**<br>O=C(C1=CC(OC)=C2C(O)=C(C(C)=O)C=C(O)C2=C1)OC	SK–019	290.27	93.07
**Methyl, 6-acetyl-4- methoxy-5,7,8-trihydroxynaphthalene-2-carboxylate**<br>O=C(C1=CC(OC)=C2C(O)=C(C(C)=O)C(O)=C(O)C2=C1)OC	SK–020	306.27	113.29

**Stigmasta-4,6,8(14), 22-tetraen-3-one**<br>CC[C@@H](C(C)C)/C=C/[C@@H](C)[C@H]1CCC2=C3C=CC4=CC(CC[C@]4(C)[C@@]3([H])CC[C@]12C)=O	SK–021	406.65	17.07
**Stigmast-4-ene-3-one**<br>CC[C@@H](C(C)C)CC[C@@H](C)[C@H]1CC[C@@]2([H])[C@]3([H])CCC4=CC(CC[C@]4(C)[C@@]3([H])CC[C@]12C)=O	SK–022	412.70	17.07
**(*22E,24R*)-stigmasta-5,7,22-trien-3-beta-ol**<br>CC[C@H](C(C)C)/C=C/[C@@H](C)[C@H]1CC[C@@]2([H])C3=CC=C4C[C@@H](O)CC[C@]4(C)[C@@]3([H])CC[C@]12C	SK–023	410.69	20.23
**(*S*)-6, 8-dimethoxy-3-methylisochroman-1-one**<br>O=C1O[C@@H](C)CC2=C1C(OC)=CC(OC)=C2	SK–024	222.24	44.77
**Lovastatin**<br>CC[C@H](C)C(O[C@H]1C[C@@H](C)C=C2C=C[C@H](C)[C@H](CC[C@H](C[C@@H](O)C3)OC3=O)[C@@]12[H])=O	SK–025	404.55	72.84
**3-hydroxy-5-[[4-hydroxy-3-(3-methyl-2-buten-1-yl)phenyl]methyl]-4-(4-hydroxyphenyl)- 2(5H)-furanone**<br>O=C1OC(CC2=CC=C(O)C(C/C=C(C)\C)=C2)C(C3=CC=C(O)C=C3)=C1O	SK–026	366.41	86.99
**Aspergillamide A**<br>CC(C)C[C@H](NC(C)=O)C(N([C@@H](CC1=CC=CC=C1)C(N/C=C\C2=CNC3=C2C=CC=C3)=O)C)=O	SK–027	474.61	94.60
**Aspergillamide B**<br>CC(C)C[C@H](NC(C)=O)C(N([C@@H](CC1=CC=CC=C1)C(N/C=C/C2=CNC3=C2C=CC=C3)=O)C)=O	SK–028	474.61	94.30
**Aspergillamide C**<br>CC[C@H](C)[C@H](NC(C)=O)C(N([C@@H](CC1=CC=C(O)C=C1)C(N/C=C\C2=CNC3=C2C=CC=C3)=O)C)=O	SK–029	490.60	114.52
**Aspergillamide D**<br>CC[C@H](C)[C@H](NC(C)=O)C(N([C@@H](CC1=CC=C(O)C=C1)C(N/C=C/C2=CNC3=C2C=CC=C3)=O)C)=O	SK–030	490.60	114.52
**Methyl, 2-(3-formyl-4-hydroxybenzyl)-4-hydroxy-3-(4-hydroxyphenyl)-5-oxo-2,5-dihydrofuran-2-carboxylate**<br>O=C1OC(CC2=CC=C(O)C(C=O)=C2)(C(OC)=O)C(C3=CC=C(O)C=C3)=C1O	SK–031	384.34	130.37
**Methyl, 3,4,5-trimethoxy-2-(2- (nicotinamido)benzamido) benzoate**<br>O=C(OC)C1=CC(OC)=C(OC)C(OC)=C1NC(C2=CC=CC=C2NC(C3=CN=CC=C3)=O)=O	SK–032	465.46	125.09
**Kigelin**<br>O=C1C2=C(C=C(OC)C(OC)=C2O)C[C@@H](C)O1	SK–033	238.24	65.00
**Questin**<br>O=C1C2=C(C=C(O)C=C2OC)C(C3=CC(C)=CC(O)=C13)=O	SK–034	284.27	83.83
**5-[(3,4-dihydro-2,2-dimethyl-2H-1-benzopyran-6-yl)- methyl]-3-hydroxy-4-(4-hydroxyphenyl)-2(5H)-furanone**<br>O=C1OC(CC2=CC=C3C(CCC(C)(C)O3)=C2)C(C4=CC=C(O)C=C4)=C1O	SK–035	366.41	76.00
**4-(4-hydroxyphenyl)-5- (4-hydroxyphenylmethyl)-2-hydroxyfuran-2-one**<br>OC1OC(CC2=CC=C(O)C=C2)C(C3=CC=C(O)C=C3)=C1	SK–036	284.31	69.92

**Butyrolactone I**<br>O=C([C@](C(C1=CC=C(O)C=C1)=C2O)(CC3=CC=C(O)C(C/C=C(C)\C)=C3)OC2=O)OC	SK–037	424.4	113.29
**Butyrolactone II**<br>O=C([C@](C(C1=CC=C(O)C=C1)=C2O)(CC3=CC=C(O)C=C3)OC2=O)OC	SK–038	356.33	113.29
**Isobutyrolactone II**<br>CO[C@]1(C(=C(C(=O)O1)O)C2=CC=C(C=C2)O)CC3=CC=C(C=C3)O	SK–039	328.32	96.22
**4-*O*-Demethylisobutyrolactone II**<br>C1=CC(=CC=C1C[C@@]2(C(=C(C(=O)O2)O)C3=CC=C(C=C3)O)O)O	SK–040	314.29	107.22
**Butyrolactone III**<br>O=C([C@](C(C1=CC=C(O)C=C1)=C2O)(CC3=CC=C(O)C(CC4OC4(C)C)=C3)OC2=O)OC	SK–041	440.45	125.82
**Butyrolactone IV**<br>CC(C)([C@H]1CC2=C(O1)C=CC(=C2)C[C@@]3(C(=C(C(=O)O3)O)C4=CC=C(C=C4)O) C(=O)OC)O	SK–042	440.45	122.53
**Butyrolactone V**<br>CC1([C@H](CC2=C(O1)C=CC(=C2)C[C@@]3(C(=C(C(=O)O3)O)C4=CC=C(C=C4)O)C(=O) OC)O)C	SK–043	440.45	122.53
**Butyrolactone VI**<br>O=C([C@](C(C1=CC=C(O)C=C1)=C2O)(CC3=CC=C(O)C(C[C@H](O)C(C)(O)C)=C3)OC2=O) OC	SK–044	458.46	153.75
**Butyrolactone VII**<br>CCOC(=O)[C@]1(C(=C(C(=O)O1)O)C2=CC=C(C=C2)O)CC3=CC(=C(C=C3)O)CC=C(C)C	SK–045	438.48	113.29
**Butyrolactone VIII**<br>CC(=CCC1=C(C=CC(=C1)CC2=C(C(OC2=O)(C(=O)OC)O)C3=CC=C(C=C3)O)O)C	SK–046	424.45	113.29
**Emodin**<br>O=C1C2=C(C=C(C)C=C2O)C(C3=CC(O)=CC(O)=C13)=O	SK–047	270.24	94.83
**Camptothecin**<br>O=C1C2=C(C=C(O)C=C2OC)C(C3=CC(C)=CC(O)=C13)=O	SK–048	284.27	83.83
**Terremide A**<br>O=C(OC)C1=CC=CC(O)=C1NC(C2=CC=CC=C2NC(C3=CC=CN=C3)=O)=O	SK–049	391.38	117.62
**Terremide B**<br>O=C(OC)C1=CC=CC(O)=C1N2C(C3=CC=CN=C3)=NC4=C(C=CC=C4)C2=O	SK–050	373.37	94.32
**Terremide C**<br>O=C(OC)C1=CC(OC)=C(OC)C(OC)=C1N2C(C3=CC=CN=C3)=NC4=C(C=CC=C4)C2=O	SK–051	447.45	101.80
**Terrelactone A**<br>O=C([C@](C(C1=CC=C(OC)C=C1)=C2O)(CC3=CC(C/C=C(C)\C)=C(O)C(OC)=C3)OC2=O)OC	SK–052	468.50	111.53
**Aspulvinone B**<br>O=C(C(C1=CC(CCC(C)(C)O2)=C2C=C1)=C/3O)OC3=C/C4=CC=C(O)C(C/C=C(C)\C)=C4	SK–053	432.52	79.90
**Aspulvinone C**<br>CC1(CCC2=CC(=C(C=C2O1)O)/C=C\3/C(=C(C(=O)O3)C4=CC5=C(C=C4)OC(CC5)(C)C)O)C	SK–054	448.51	89.14
**Aspulvinone E**<br>O=C(C(C1=CC=C(O)C=C1)=C/2O)OC2=C/C3=CC=C(O)C=C3	SK–055	296.28	90.89
**Aspulvinone D**<br>CC(=CCC1=C(C=CC(=C1)/C=C\2/C(=C(C(=O)O2)C3=C(C=C4C(=C3)CCC(O4)(C)C)O)O)O)C	SK–056	448.51	100.13
**Isoaspulvinone E**<br>O=C(C(C1=CC=C(O)C=C1)=C/2O)OC2=C\C3=CC=C(O)C=C3	SK–057	296.28	90.89
**Aspulvinone F**	SK–058	464.51	109.36

O=C(C(C1=CC(CCC(C)(C)O2)=C2C=C1O)=C/3O)OC3=C/C4=CC5=C(OC(C(C)(O)C)C5)C=C4			
**Aspulvinone G**<br>O=C(C(C1=CC=C(O)C=C1O)=C/2O)OC2=C/C3=CC=C(O)C=C3	SK–059	312.28	111.12
**Aspulvinone H**<br>O=C(C(C1=CC=C(O)C(C/C=C(C)\C)=C1)=C/2O)OC2=C/C3=CC=C(O)C(C/C=C(C)\C)=C3	SK–060	432.52	90.89
**Rubrolide S**<br>O=C(C=C/1C2=CC=C(O)C=C2)OC1=C/C3=CC(CCC(C)(C)O4)=C4C=C3	SK–061	348.40	59.67
**Rubrolide R**<br>O=C(C=C/1C2=CC=C(O)C=C2)OC1=C/C3=CC=C(O)C(C/C=C(C)\C)=C3	SK–062	348.40	70.67
**Aspulvinone R**<br>O=C(C(C1=CC(CCC(C)(O)C)=C(O)C=C1O)=C/2O)OC2=C/C3=CC=C(O)C(C/C=C(C)\C)=C3	SK–063	466.53	131.35
**Aspulvinone V**<br>O=C(C(C1=CC(C=CC(C)(C)O2)=C2C(C/C=C(C)\C)=C1)=C/3O)OC3=C/C4=CC=C(O)C<br>(C/C=C(C)\C)=C4	SK–064	498.62	79.90
**Aspulvinone W**<br>O=C(C(C1=CC(C/C=C(C)/C)=C(O)C=C1)=C/2O)OC2=C/C3=CC=C(CC(C(C)(C)O)O4)C4=C3	SK–065	448.51	100.13
**Aspulvinone X**<br>O=C(C(C1=CC(CCC(C)(C)O2)=C2C=C1)=C/3O)OC3=C/C4=CC=C(CC(C(C)(C)O)O5)C5=C4	SK–066	448.51	89.14
**Kodaistatin A**<br>O=C(C(C1=CC(O)=C(O)C=C1C2=C(CC(/C=C/C(C)=C/C(C)CC)=O)C(C(O)C2(C(C)=O)O)=O)<br>=C/3O)OC3=C/C4=CC=C(O)C=C4	SK–067	630.65	202.79
**Kodaistatin C**<br>O=C(C(C1=CC(O)=C(O)C=C1C2=C(CC(/C=C/C(C)=C/C(C)CC)=O)C(C(O)C2(C(C)=O)O)=O)<br>=C/3O)OC3=C/C4=CC=C(O)C(O)=C4	SK–068	646.64	223.02
**Versicolactone G**<br>O=C([C@](C(C1=CC=CC=C1)=C2O)(CC3=CC=C(O)C(CCC(C)(OC)C)=C3)OC2=O)OC	SK–069	440.49	102.30
**(+)-Terrein**<br>O=C1C=C(/C=C/C)[C@H](O)[C@H]1O	SK–070	154.16	57.53
**Butyrolactone I, 2-sulfate**<br>O=C([C@](C(C1=CC=C(O)C=C1)=C2OS(=O)(O)=O)(CC3=CC=C(O)C(C/C=C(C)\C)=C3)<br>OC2=O)OC	SK–071	504.51	156.67
**Butyrolactone I, 4``-Sulfated**<br>O=C([C@](C(C1=CC=C(O)C=C1)C2=O)(CC3=CC=C(OS(=O)(O)=O)C(C/C=C(C)\C)=C3)<br>OC2=O)OC	SK–072	504.51	153.51
**7``-Hydroxybutyrolactone III**<br>O=C([C@](C(C1=CC=C(O)C=C1)=C2O)(CC3=CC=C(O)C(C(O)C4OC4(C)C)=C3)OC2=O)OC	SK– 073	456.44	146.05
**(+)-3′,3′-di-(dimethylallyl)-butyrolactone II**<br>CC(=CCC1=C(C=CC(=C1)C[C@@]2(C(=C(C(=O)O2)O)C3=CC(=C(C=C3)O)CC<br>=C(C)C)C(=O)OC)O)C	SK– 074	492.57	113.29
**14α-hydroxyergosta-4,7,22-triene-3,6-dione**<br>C[C@H](C(C)C)/C=C/[C@@H](C)[C@H]1CC[C@@]2(O)C3=CC(C4=CC(CC[C@]4(C)<br>[C@@]3([H])CC[C@]12C)=O)=O	SK–075	424.62	54.37
**Asterrelenin**	SK–076	431.49	89.94

O=C(NC1=CC=CC=C21)[C@@]3(O)C[C@@]4(C(C=C)(C)C)C5=CC=CC=C5N(C(C)=O)[C@@]4([H])N3C2=O			
**(*3E,7E*)-4,8-dimethylundeca-3,7-diene-1,11-diol**<br>OCC/C=C(C)/CC/C=C(C)/CCCO	SK–077	212.33	40.46
**Monacolin L**<br>O=C1O[C@H](CC[C@H]2[C@@H](C)C=CC3=C[C@H](C)CC[C@]23[H])C[C@@H](O)C1	SK–078	304.43	46.53
**Pulvinic acid**<br>C1=CC=C(C=C1)C2=C(/C(=C(/C3=CC=CC=C3)\C(=O)O)/OC2=O)O	SK–079	308.28	87.74
**Terrephenol A**<br>CC(=CCC1=C(C(=CC(=C1)C[C@@]2(C(=C(C(=O)O2)O)C3=CC=C(C=C3)O)C(=O)OC)OC) O)C	SK–080	454.48	122.53
**Terrephenol B**<br>CC(=CCC1=C(C=CC(=C1O)C[C@@]2(C(=C(C(=O)O2)O)C3=CC=C(C=C3)O)C(=O)OC)OC)C	SK–081	454.48	122.53
**Terrephenol C**<br>O=C([C@](C(C1=CC=C(OC)C=C1)=C2O)(CC3=CC(C/C=C(C)/C)=C(O)C(OC)=C3)OC2=O)OC	SK–082	468.50	111.53
**Terrenolide S**<br>CCOC1=C([C@](OC1=O)(CC2=CC=C(C=C2)O)C(=O)OC)C3=CC=C(C=C3)O	SK–083	384.38	102.30
**Xenofuranone A**<br>COC1=C(C(OC1=O)CC2=CC=CC=C2)C3=CC=CC=C3	SK–084	280.32	35.54
**Xenofuranone B**<br>C1=CC=C(C=C1)CC2C(=C(C(=O)O2)O)C3=CC=CC=C3	SK–085	266.30	46.53
**Luteoride E**<br>O=C(OC)/C(CC1=CNC2=C1C=CC=C2C/C=C(C)\C)=N/O	SK–086	300.36	74.69
**3-hydroxy-5-(4-hydroxybenzyl)-4-(4-hydroxyphenyl)furan-2(5H)-one**<br>O=C1C(O)=C(C2=CC=C(O)C=C2)C(CC3=CC=C(O)C=C3)O1	SK–087	298.29	86.99
**5-[(3,4-Dihydro-2,2-dimethyl-2H-1-benzopyran-6-yl)-methyl]-3- hydroxy-4-(4-hydroxyphenyl)-2(5H)-furanone**<br>O=C1OC(CC2=CC=C3C(CCC(C)(C)O3)=C2)C(C4=CC=C(O)C=C4)=C1O	SK–088	366.41	76.00
**Arisugasin A**<br>O=C1[C@]2(C)[C@@]3(O)CC4=C(C=C(C5=CC=C(OC)C(OC)=C5)OC4=O)O[C@]3(C)CC[C@@]2(O)C(C)(C)C=C1	SK–089	496.56	115.44
**Terreulactone A**<br>C[C@@]12CC[C@]34[C@@]([C@]1(CC5=C(O2)C=C(OC5=O)C6=CC=C(C=C6)OC)O)(C(=O)[C@](C3(C)C)(C(=O)O4)OC)C	SK–090	510.54	121.52
**Terreulactone B**<br>C[C@@]12CC[C@@]3([C@@]([C@]1(CC4=C(O2)C=C(OC4=O)C5=CC=C(C=C5)OC)O)(C=CC(=O)C3(C)C)C)O	SK–091	466.53	106.21
**Terreulactone C**<br>C[C@]12CCC(=O)C([C@@]1(CC[C@@]3([C@@]2(CC4=C(O3)C=C(OC4=O)C5=CC=C(C=C5)OC)O)C)O)(C)C	SK–092	468.55	103.21
**Terreulactone D**<br>C[C@@]12CC[C@@]3([C@@]([C@]1(CC4=C(O2)C=C(OC4=O)C5=CC(=C(C(=C5)OC)OC)OC)O)(C(=O)C=C(C3(C)C)OC)C)O	SK–093	556.61	133.91

**Psoralenone**<br>O=C1C(C2=CC(C[C@H](O)C(C)(C)O3)=C3C=C2)=COC4=C1C=C(OC)C(OC)=C4	SK–094	382.41	78.14
**Aspulvinone I**<br>CC(=CCC1=C(C=CC(=C1)/C=C\2/C(=C(C(=O)O2)C3=CC=C(C=C3)O)O)O)C	SK–095	364.40	90.89
**Blasticidin S**<br>CN(CC[C@@H](CC(=O)N[C@H]1C=C[C@@H](O[C@@H]1C(=O)O)N2C=CC(=NC2=O)N)N)C(=N)N	SK–096	422.45	215.69
**Terreic acid (5,6-epoxy-3-hydroxy-p-toluquinone)**<br>CC1=C(C(=O)[C@H]2[C@@H](C1=O)O2)O	SK–097	154.12	66.90
**Novobenzomalvin D**<br>O=C(NCC(C1=CC=CC=C1)=O)C2=CC=CC=C2N3C(NC4=C(C=CC=C4)C3=O)=O	SK–098	399.41	101.04
**Astalluminoxide**<br>CC1=NC(OC)=C(CC2=CNC3=C(C/C=C(C)\C)C=CC=C23)N([O-])C1=O.CC4=NC(OC)=C(CC5=CNC6=C(C/C=C(C)\C)C=CC=C56)N([O-])C4=O.CC7=NC(OC)=C(CC8=CNC9=C(C/C=C(C)\C)C=CC=C89)N([O-])C7=O.[Al+3]	SK–099	1083.48	255.12
**Astechrome**<br>CC1=NC(OC)=C(CC2=CNC3=C(C/C=C(C)\C)C=CC=C23)N(O)C1=[OH+].CC4=NC(OC)=C(CC5=CNC6=C(C/C=C(C)\C)C=CC=C56)N(O)C4=[OH+].CC7=NC(OC)=C(CC8=CNC9=C(C/C=C(C)\C)C=CC=C89)N(O)C7=[OH+].[Fe]	SK–100	1119.11	263.02
**3-hydroxy-5-(4-hydroxybenzyl)-4-(4-hydroxyphenyl)furan-2(5H)-one**<br>O=C1OC(CC2=CC=C(O)C=C2)C(C3=CC=C(O)C=C3)=C1O	SK–101	298.29	86.99
**3-(p-hydroxyphenyl)-4-hydroxy-S-(p-hydroxybenzylidene)-2(5H)-furanone**<br>O=C(C(C1=CC=C(O)C=C1)=C/2O)OC2=C\C3=CC=C(O)C=C3	SK–102	296.28	90.89
**Versicolactone B**<br>C/C/1=C/C[C@@H]([C@H]2C[C@@H](CC[C@@H]1O)C(=O)O2)C(=C)C	SK–103	250.34	46.53
**Bisdethiodi(methylthio)acetylaranotin**<br>CC(OC(C=COC=C1C[C@@]23SC)C1N3C([C@@]4(SC)CC5=COC=CC(OC(C)=O)C5N4C2=O)=O)=O	SK–104	534.61	111.70
**Terrusnolide A**<br>CC(=CCC1=C(C=CC(=C1)CC(=O)CC2=CC=C(C=C2)O)O)C	SK–105	310.39	57.53
**(+)-Geodin**<br>CC1=C(C(=C2C(=C1Cl)O[C@]3(C2=O)C(=CC(=O)C=C3OC)C(=O)OC)O)Cl	SK–106	399.18	99.14
**Botryosphaerin B**<br>C[C@]12CCC[C@]([C@@H]1C=C[C@]3([C@@H]2CC(=O)O3)CO)(C)C(=O)O	SK–107	294.35	83.83
**Botryosphaerin F**<br>C[C@]12CCC[C@]3([C@@H]1[C@@H]([C@H]([C@H]4C2=CC(=O)OC4)O)OC3=O)C	SK–108	292.33	72.84
**13,14,15,16-tetranorlabd-7-ene-19,6b:12,17-diolide**<br>O=C(OCC1=C[C@@]2([H])O3)CC1[C@@]([C@@]24[H])(C)CCC[C@]4(C)C3=O	SK–109	276.33	52.61
**LLZ1271b**<br>O=C([C@@]1(C)CCC[C@]2(C)[C@@H](CC(O)=O)C(C(O)C[C@@]12[H])=C)O	SK–110	296.36	94.83
**Anhydrojavanicin**<br>CC1=CC2=C(C(=C3C(=O)C=C(C(=O)C3=C2O1)OC)O)C	SK–111	272.26	76.74
**8-*O*-methylbostrycoidin**	SK–112	299.28	85.73

CC1=CC2=C(C=N1)C(=O)C3=C(C2=O)C(=C(C=C3OC)OC)O			
**3β,5α-dihydroxy-(22E,24R)-ergosta-7,22-dien-6-one** O=C1[C@@]2(O)C[C@@H](O)CC[C@]2(C)[C@@]3(C)CC[C@]4(C)[C@@H]([C@@H](/C=C/[C@H](C)C(C)C)C)CC[C@@]4([H])C3=C1	SK–113	442.68	57.53
**3β,5α,14α-trihydroxy-(22E,24R)-ergosta-7,22-dien-6-one** O=C1[C@@]2(O)C[C@@H](O)CC[C@]2(C)[C@@]3(C)CC[C@]4(C)[C@@H]([C@@H](/C=C/[C@H](C)C(C)C)C)CC[C@@]4(O)C3=C1	SK–114	458.68	77.75
**NGA0187** C[C@H](/C=C/[C@H](C)C(C)C)[C@H]1[C@H](C[C@@H]2[C@@]1(C[C@@H](C3C2[C@H](C(=O)[C@@H]4[C@@]3(CC[C@@H](C4)O)C)O)O)C)OC(=O)C	SK–115	504.71	104.06
**Beauvericin** CC(C)[C@@H]1C(=O)N([C@H](C(=O)O[C@@H](C(=O)N([C@H](C(=O)O[C@@H](C(=O)N([C@H](C(=O)O1)CC2=CC=CC=C2)C)C(C)C)CC3=CC=CC=C3)C)C(C)C)CC4=CC=CC=C4)C	SK–116	783.96	139.84
**Terrstatin A** C[C@H]1C=CC2=C[C@H](CC[C@@H]2[C@H]1CCC(=O)OC)CO	SK–117	264.37	46.83
**Terrstatin B** C[C@H]1C=CC2=C[C@@H](CC[C@@H]2[C@H]1CCC(=O)OC)CO	SK–118	264.37	46.53
**(+)-Asperteretone F** CC(=CCC1=C(C=CC(=C1)C(=O)C2=C(C(=O)O[C@H]2OC)C3=CC=C(C=C3)O)O)C	SK–119	394.42	93.07
**(−)-Asperteretone F** CC(=CCC1=C(C=CC(=C1)C(=O)C2=C(C(=O)O[C@@H]2OC)C3=CC=C(C=C3)O)O)C	SK–120	394.42	93.07
**Sulochrin** CC1=CC(=C(C(=C1)O)C(=O)C2=C(C=C(C=C2OC)O)C(=O)OC)O	SK–121	332.31	113.29
**(2'*R*)-Westerdijkin A** O=C(OC)CC1=CC=C(OC[C@H](O)C(C)=C)C=C1	SK–122	250.29	55.77
**Methyl, 4-hydroxyphenylacetate** O=C(OC)CC1=CC=C(O)C=C1	SK–123	166.18	46.53
**Ampelopyrone** CC1=C(C=C(OC1=O)CC(C)OC(=O)C)O	SK–124	226.23	76.74
**Sporogen-AO 1** C[C@H]1[C@@H](CCC2=CC(=O)[C@]3([C@@H]([C@]12C)O3)C(=C)C)O	SK–125	248.32	49.83
**Asperdemin** CC1=CC2=C(C[C@H]3[C@](O2)(C[C@H]([C@@H]4[C@@]3([C@H](CC(=O)OC4(C)C)O)C)O)C)C(=O)O1	SK–126	392.45	106.21
**(*S*)-(+)-11-dehydrosydonic acid** CC(=C)CCC[C@@](C)(C1=C(C=C(C=C1)C(=O)O)O)O	SK–127	264.32	77.75
**Asperversin E** CC1=CC2=C(C[C@H]3[C@](O2)(C[C@H]([C@@H]4[C@@]3([C@H](CC(=O)OC4(C)C)O)C)OC(=O)C)C)C(=O)O1	SK–128	434.49	112.28
**Asperversin G** CC1=CC2=C(C[C@H]3[C@](O2)(C[C@H]([C@@H]4[C@@]3(C=CC(=O)C4(C)C)C)OC(=O)C)C)C(=O)O1	SK–129	40.47	82.82

**Asperterone B**<br>O=C(C(CC1=CC=C(O)C(CCC(C)(O)C)=C1)=C2C3=CC=C(O)C=C3)OC2=O	SK–130	382.41	107.97
**Asperterone C**<br>O=C(C(CC1=CC=C2C(CCC(C)(C)O2)=C1)=C3C4=CC=C(O)C=C4)OC3=O	SK–131	364.40	76.74
**12β,15α,25,28-tetrahydroxyergosta-4,6,8(14),22-tetraen-3-one**<br>CC(C)(O)[C@@H](CO)/C=C/[C@@H](C)[C@H]1CC(O)C2=C3C=CC4=CC(CC[C@]4(C)<br>[C@@]3([H])CC(O)[C@]12C)=O	SK–132	456.62	97.98
**Terrequinone A**<br>CC(=CCC1=C(C(=O)C(=O)C(=C1O)C2=C(NC3=CC=CC=C32)C(C)(C)C=C)C4=CNC5 =CC=CC=C54)C	SK–133	490.60	85.95
**Oleic acid**<br>CCCCCCCC/C=C\CCCCCCCC(=O)O	SK–134	282.5	37.30
**Terrefuranone**<br>O=C1[C@](C)(/C=C/C=C/CC)OC(CC(O)C)=C1	SK–135	236.31	46.53
**Terremutin hydrate**<br>O=C1C(C)=C(O)[C@H](O)[C@@H](O)[C@@H]1O	SK–136	174.15	97.98
**Asterric acid**<br>O=C(O)C1=C(OC2=C(C(OC)=O)C=C(O)C=C2OC)C=C(C)C=C1O	SK–137	348.31	122.53
**Dihydroerdin**<br>O=C(O)C1=CC(O)=CC(OC)=C1C(C2=C(O)C(Cl)=C(C)C(Cl)=C2O)=O	SK–138	387.17	124.29
**Sulochrin**<br>O=C(OC)C1=CC(O)=CC(OC)=C1C(C2=C(O)C=C(C)C=C2O)=O	SK–139	332.31	113.29
**Taxol**<br>O=C(O[C@@H]([C@]1([H])[C@]2(OC(C)=O)CO[C@]2([H])C[C@H](O)[C@@]31C)<br>[C@](C4(C)C)(O)C[C@H](OC([C@H](O)[C@@H](NC(C5=CC=CC=C5)=O)C6=CC<br>=CC=C6)=O)C(C)=C4[C@@H](OC(C)=O)C3=O)C7=CC=CC=C7	SK–143	853.92	221.31
**3,6-dihydroxytoluquinone**<br>O=C1C(C)=C(O)C(C=C1O)=O	SK–144	154.12	74.60
**Terreusinone**<br>O=C(C1=C2C=C([C@H](O)C(C)C)N1)C3=C(NC([C@H](O)C(C)C)=C3)C2=O	SK–145	330.38	106.18
**Citreoviridin**<br>O=C1C=C(OC)C(C)=C(/C=C/C=C/C=C/C(C)=C/[C@]2(C)O[C@H](C)[C@](C)(O)[C@H]2O)O1	SK–146	402.49	89.14
**Asperlide A**<br>CC1(C(CC2=C(O1)C=CC(=C2)C[C@@]3(C(=C(C(=O)O3)O)C4=CC=CC=C4)C(=O)OC)O)C	SK–147	424.45	102.30
**Asperlide B**<br>O=C([C@](C(C1=CC=CC=C1)=C2O)(CC3=CC4=C(OC(C(C)(O)C)C4)C=C3)OC2=O)OC	SK–148	424.45	102.30
**Asperlide C**<br>O=C1C(O)=C(C2=CC=C(O)C=C2)[C@@H](CC3=CC4=C(OC(C(C)(O)C)C4)C=C3)O1	SK–149	382.41	96.22
**Asperterpene D**<br>O=C([C@](OC([C@]1([H])[C@@]2(C)C(C(O)=C3C4(C)C)=O)=O)(C)C([C@]1(C)C([C@]<br>5([H])O[C@]25[C@]3(C)CCC4=O)=C)=O)OC	SK–150	486.52	136.58
**Asperterpen E**<br>O=C([C@@](C([C@]1(C)C(C[C@]2([H])[C@@]([C@H](O)C([C@@]3([H])C(C)(C)C(CC[C@]<br>32C)=O)=O)(C)[C@]14[H])=C)=O)(C)OC4=O)OC	SK–151	474.55	124.05

**Asperterpene F**<br>C[C@H]1C([C@@]2(C)[C@@H]([C@]3(C)[C@H]([C@]4(C)CCC(C(C)(C)[C@@H]4C([C@H]3O)=O)=O)CC2=C)C(O1)=O)=O	SK–152	416.51	97.75
**Asperterpene G**<br>O=C([C@@](C([C@]1(C)C(C[C@]2([H])[C@@]([C@H](O)C([C@]3([H])C(C)(C)C(CC[C@]32C)=O)=O)(C)[C@@]14[H])=C)=O)(C)OC4=O)OC	SK–153	474.55	124.05
**Asperterpene H**<br>O=C([C@@](C([C@]1(C)C(C[C@]2([H])[C@@]([C@H](O)C([C@]3([H])C(C)(C)C(CC[C@]32C)=O)=O)(C)[C@]14[H])=C)=O)(C)OC4=O)OCC	SK–154	488.58	124.05
**Asperterpene I**<br>O=C([C@H]1[C@](C)(C([C@](C)(O)C(OC)=O)=O)C(C[C@@]2([H])[C@]3(C)CC[C@@H](O)C(C)(C)[C@@]3([H])C([C@@H](O)[C@]12C)=O)=C)OC	SK–155	508.61	147.44
**Asperterpene J**<br>O=C([C@H]1[C@](C)(C([C@](C)(O)C(OC)=O)=O)C(C[C@@]2([H])[C@]3(C)CCC(C(C)(C)[C@]3([H])C([C@@H](O)[C@]12C)=O)=O)=C)OC	SK–156	506.59	144.28
**Asperterpene K**<br>O=C([C@H]1[C@](C)(C([C@](C)(O)C(OC)=O)=O)C(C[C@@]2([H])[C@]3(C)CCC(C(C)(C)[C@@]3([H])C(C[C@]12C)=O)=O)=C)OC	SK–157	490.59	124.05
**Asperterpene L**<br>O=C([C@H]1[C@](C)(C([C@](C)(O)C(OC)=O)=O)C(C[C@@]2([H])[C@]3(C)CCC(C(C)(C)C3=C(O)C([C@]12C)=O)=O)=C)OC	SK–158	504.58	144.28
**Asperterpene M**<br>C[C@H]1C([C@@]2(C(OC)=O)[C@@]3(C)CC([C@H]4[C@](CCC(C4(C)C)=O)(C)[C@@H]3CC([C@@]2(C)C(O1)=O)=C)=O)=O	SK–159	458.55	103.82
**Terretonin A**<br>O=C([C@](C([C@@]1(C)C(C[C@@]2([H])[C@](C(C(O)=C3C(C)(C)C(CC[C@@]32C)=O)=O)(C)[C@@]14[H])=C)=O)(C)OC4=O)OC	SK–160	472.53	124.05
**Terretonin B**<br>O=C([C@](C([C@@]1(C)C(C[C@@]2(O)[C@](C(C(O)=C3C(C)(C)C(CC[C@@]32C)=O)=O)(C)[C@@]14[H])=C)=O)(C)OC4=O)OC	SK–161	488.53	144.28
**Terretonin D**<br>O=C([C@](C([C@@]1(C)C(C[C@@]2([H])[C@]([C@@H](O)C([C@@]3([H])C(C)(C)C(CC[C@@]32C)=O)=O)(C)[C@@]14[H])=C)=O)(C)OC4=O)OC	SK–162	474.55	124.05
**Terretonin G**<br>O=C([C@H]1[C@](C)(C([C@](C)(O)C(OC)=O)=O)C(C[C@@]2([H])[C@]3(C)CCC(C(C)(C)[C@@]3([H])C([C@@H](O)[C@]12C)=O)=O)=C)OC	SK–163	506.59	144.28
**Emestrins B**<br>O=C([C@@](SC)(CC1=CC=CC=C1)N2)N[C@](SC)(CC3=CC=CC=C3)C2=O	SK–164	386.54	58.20
**Emestrins C**<br>O=C([C@](SC)(CC1=CC=CC=C1)N2)N[C@@](SC)(CC3=CC=CC=C3)C2=O	SK–165	386.54	58.20
**Emestrins L**<br>CC(O[C@H]([C@@]1([H])N2C([C@](SC)(CC3=CC=CC=C3O)N4)=O)C=COC=C1C[C@@]2 (SC)C4=O)=O	SK–166	476.58	105.17

**Emestrins M**<br>O=C([C@](SC)(CC1=CC=CC=C1O)N2)N3[C@]4([H])[C@@H](O)C=COC=C4C[C@@]3(SC)C2=O	SK–167	434.54	99.10
**Pseurotin A3**<br>O=C1C(C)=C([C@@H](O)[C@@H](O)/C=C\CC)O[C@]12C(N[C@](OC)(C(C3=CC=CC=C3)=O)[C@@H]2O)=O	SK–168	431.44	142.39
**4-hydroxy-3-(3-methylbut-2-enyl)benzaldehyde**<br>O=CC1=CC=C(O)C(C/C=C(C)\C)=C1	SK–169	190.24	37.30
**5-*N*-acetylardeemin**<br>O=C(N1[C@@H]2C)C3=CC=CC=C3N=C1[C@]4([H])C[C@@]5(C(C=C)(C)C)C6=CC=CC=C6N(C(C)=O)[C@@]5([H])N4C2=O	SK–170	468.56	75.52
**Gillusdin**<br>O=C(C(C12OC3=C(C(C)=C(Cl)C(O)=C3Cl)C1=O)=CC(C(O)=C2OC)=O)OC	SK–171	415.18	119.37
**Terrelumamide A**<br>O=C(OC)C1=CC=CC=C1NC([C@@H](NC(C2=CN=C3N(C)C(NC(C3=N2)=O)=O)=O)[C@H](O)C)=O	SK–172	456.42	185.38
**Terrelumamide B**<br>O=C(OC)C1=CC=CC=C1NC([C@@H](NC(C2=CN=C3N(C)C(NC(C3=N2)=O)=O)=O)CO)=O	SK–173	442.39	185.38
**Terreinol**<br>O=C1C2(OCCC2)OCC3=C1C=C(O)C(C)=C3O	SK–174	250.25	76.00
**Asterriquinone**<br>O=C1C(O)=C(C2=CN(C(C=C)(C)C)C3=C2C=CC=C3)C(C(O)=C1C4=CN(C(C=C)(C)C)C5=C4C=CC=C5)=O	SK–175	506.60	84.47
**Isoasterriquinone**<br>O=C1C(O)=C(C2=CNC3=C2C=CC(C/C=C(C)\C)=C3)C(C(O)=C1C4=CNC5=C4C=CC(C/C=C(C)\C)=C5)=O	SK–176	506.60	106.18
**Terremutin**<br>O=C1[C@@]2([H])O[C@@]2([H])[C@H](O)C(O)=C1C	SK–177	156.14	70.06
**Citrinin**<br>O=C(C1=C(O)C(C)=C2[C@H](C)[C@@H](C)OC=C2C1=O)O	SK–178	250.25	83.83
**10-phenyl-[12]-cytochalasins Z16**<br>O=C(C/C=C(C)\C([C@@H](C)C/C=C/[C@@]1([H])[C@H](O)C([C@@H](C)[C@@]2([H])[C@H](CC3=CC=CC=C3)N4)=C)=O)O[C@]12C4=O	SK–179	463.57	92.70
**Rosellichalasin**<br>O=C(C/C=C(C)\C(C(C)C/C=C/C1C2OC2(C)C(C)C3C(CC4=CC=CC=C4)N5)=O)OC13C5=O	SK–180	463.57	85.00
**Cytochalasin E**<br>O=C(O/C=C/[C@@](C)(O)C([C@@H](C)C/C=C/[C@@]1([H])[C@]2([H])O[C@]2(C)[C@@H](C)[C@@]3([H])[C@H](CC4=CC=CC=C4)N5)=O)O[C@]13C5=O	SK–181	495.57	114.46
**Cytochalasin Z11**<br>O=C1N[C@@H](CC2=CC=CC=C2)[C@]3([H])[C@H](C)C([C@@H](O)[C@H](/C=C/C[C@H](C)C([C@@H](O)C)=O)[C@]13O)=C	SK–182	427.54	106.85
**Cytochalasin Z13**<br>O=C1N[C@@H](CC2=CC=CC=C2)[C@]3([H])C(C)=C(C)[C@@H](O)[C@H](/C=C/C[C@H](C)C([C@@H](O)C)=O)[C@]13O	SK–183	427.54	106.85

**(+)-isoterrein**<br>O=C1C=C(/C=C/C)[C@H](O)[C@@H]1O	SK–184	154.16	57.53
**4-hydroxy-3-(3-methyl-2-buten-1-yl)-benzaldehyde**<br>O=CC1=CC=C(O)C(C/C=C(C)\C)=C1	SK–185	190.24	37.30
**Gliotoxin**<br>O=C1N2[C@]3([H])[C@@H](O)C=CC=C3C[C@@]42SS[C@@]1(CO)N(C)C4=O	SK–186	326.40	81.07
**Acetylaranotin**<br>CC(OC(C=COC=C1CC23SS4)C1N3C(C54CC6=COC=CC(OC(C)=O)C6N5C2=O)=O)=O	SK–187	504.54	111.70
**Terreustoxin C**<br>C[C@H]1C([C@@]2(C)[C@@H]([C@]3(C)[C@H]([C@]4(C)CCC(C(C)(C)[C@H]4[C@@H]<br>(O)[C@H]3O)=O)CC2=C)C(O1)=O)=O	SK–188	418.53	100.90
**Terretonin N**<br>C[C@H]1C([C@@]2(C)[C@@H]([C@@]3(C)C[C@H](O)[C@H]4[C@](CC[C@H](OC(C)<br>=O)C4(C)C)(C)[C@@H]3[C@H](O)C2=C)C(O1)=O)=O	SK–189	462.58	110.14
**Astepyrazinoxide**<br>O=C1C(C)=NC(OC)=C(CC2=CNC3=C2C=CC=C3C/C=C(C)\C)N1O	SK–190	353.42	80.15
**Astepyrazinol A**<br>O=C1C(C)=NC(OC)=C(CC2=CNC3=C2C=CC=C3C/C=C(C)\C)N1	SK–191	337.42	70.78
**Aspulvinone S**<br>O=C(C(C1=CC(C/C=C(C)\C)=C(O)C=C1O)=C/2O)OC2=C/C3=CC(CCC(C)(C)O4)=C4C=C3	SK–192	448.51	100.13
**Astepyrazinol B**<br>O=C1NC2=C(C=CC=C2C/C=C(C)\C)[C@@H]1CC(N3)=C(OC)N=C(C)C3=O	SK–195	353.42	84.09
**Asperteretal D**<br>O=C1C(CC2=CC=C(O)C(C/C=C(C)\C)=C2)=C(C3=CC=C(O)C=C3)C(OC)O1	SK–196	380.44	76.00
**Asperteretal E**<br>O=C1C(CC2=CC(CCC(C)(C)O3)=C3C=C2)=C(C4=CC=C(O)C=C4)C(O)O1	SK–197	366.41	76.00
**Asperteretal F**<br>O=C1C(CC2=CC=C(O)C(C/C=C(C)/C)=C2)=C(C3=CC=C(O)C=C3)C(O)O1	SK–198	366.41	86.99
**Asperteretal G1**<br>O=C(O)[C@H](CC1=CC=C(O)C(C/C=C(C)\C)=C1)[C@@H](C2=CC=C(O)C=C2)C(OC)=O	SK–199	398.45	104.06
**Asperteretal G2**<br>O=C(O)[C@@H](CC1=CC=C(O)C(C/C=C(C)\C)=C1)[C@@H](C2=CC=C(O)C=C2)C(OC)=O	SK–200	398.45	104.06
**Asperteretal H**<br>O=C(OC)[C@H](O)[C@H](C1=CC=C(O)C=C1)C(CC2=CC=C(O)C(C/C=C(C)\C)=C2)=O	SK–201	398.45	104.06
**Asperteretal I**<br>O=C1C(CC2=CC(CC(O)C(C)(C)O3)=C3C=C2)=C(C4=CC=C(O)C=C4)C(O)O1	SK–202	382.41	96.22
**Asperteretone A**<br>O=C(O)[C@H](C1=CC=C(O)C=C1)[C@H](CC2=CC=C(C/C=C(C)\C)C(O)=C2)C(OC)=O	SK–203	398.45	104.06
**Asperteretone B**<br>O=C1C(C2=CC=C(O)C=C2)=C(CC3=CC=C(O)C(C/C=C(C)\C)=C3)[C@@H](OC)O1	SK–204	380.44	76.00
**Asperteretone C**<br>O=C1C(C2=CC=C(OC)C=C2)=C(CC3=CC4=C(OC(C(C)(O)C)C4)C=C3)[C@@H](OC)O1	SK–205	410.47	74.23

**Asperteretone D**<br>O=C1C(C2=CC=C(O)C=C2)=C(CC3=CC(CCC(C)(C)O4)=C4C=C3)[C@@H](OC)O1	SK–206	380.44	65.00
**Asperteretone E**<br>O=C1C(C2=CC=C(O)C=C2)=C(CC3=CC=C(O)C(C/C=C(C)\C)=C3)C(OC)O1	SK–207	380.44	76.00
**Amauromine B**<br>CC(O[C@]1(C[C@@]23[H])C4=CC=CC=C4N[C@@]1([H])N3C([C@]5([H])C[C@@]6(C(C=C)(C)C)C7=CC=CC=C7N[C@@]6([H])N5C2=O)=O)=O	SK–208	498.58	90.98
**Austalide N**<br>CC(O[C@@H]1C2=C(C(C)=C3COC(C3=C2OC)=O)O[C@]4(C)C[C@@H](O)[C@@]5(O6)C(C)(C)O[C@@]6(OC)CC[C@]5(C)[C@@]14[H])=O	SK–209	532.59	119.01
**(*R,E*)-3-(2,2-dimethylchroman-6-yl)-4-hydroxy-5-((2-(2-hydroxypropan-2-yl)-2,3-dihydrobenzofuran-5-yl)methylene)furan-2(5H)-one**<br>O=C(C(C1=CC(CCC(C)(C)O2)=C2C=C1)=C/3O)OC3=C\C4=CC=C(O[C@@H](C(C)(O)C)C5)C5=C4	SK–210	448.51	89.14
**Soyasapogenol B**<br>O[C@H]1CC[C@]2(C)[C@@]3([H])CC=C4[C@]5([H])CC(C)(C)C[C@@H](O)[C@]5(C)CC[C@@]4(C)[C@]3(C)CC[C@@]2([H])[C@]1(CO)C	SK–211	458.73	60.68
**Decanoic acid**<br>CCCCCCCCCC(O)=O	SK–212	172.27	37.30
**Dodecanoic acid**<br>CCCCCCCCCCCC(O)=O	SK–213	200.32	37.30
**(*9Z,12Z*)-*N*-(2-hydroxyethyl) octadeca-9,12- dienamide**<br>CCCCC/C=C\C/C=C\CCCCCCCC(NCCO)=O	SK–214	323.52	49.33
**4β-hydroxy-6,7-dimethoxyisoflavone**<br>O=C1C(C2=CC=C(O)C=C2)=COC3=C1C=C(OC)C(OC)=C3	SK–215	298.29	68.91
**Isoterreulactone A**<br>O=C1CC[C@]2(C)[C@@]3(O)CC4=C(C=C(C5=CC=C(OC)C=C5)OC4=O)O[C@]3(C)CC[C@@]2(O)C(C)(C)O1	SK–216	484.55	115.44
**12a-dehydroxyisoterreulactone A**<br>C[C@]12CCC(=O)OC([C@@]1(CC[C@@]3([C@@H]2CC4=C(O3)C=C(OC4=O)C5=CC=C (C=C5) OC)C)O)(C)C	SK–217	468.55	95.21
**Blumenol A**<br>O=C1C=C(C)[C@@](/C=C/[C@H](O)C)(O)C(C)(C)C1	SK–218	224.30	57.53
**Genistein**<br>O=C1C(C2=CC=C(O)C=C2)=COC3=C1C(O)=CC(O)=C3	SK–219	270.24	90.89
**Biochanin A**<br>O=C1C(C2=CC=C(OC)C=C2)=COC3=C1C(O)=CC(O)=C3	SK–220	284.27	79.90
**Psoralenol**<br>O=C1C(C2=CC(CC(O)C(C)(C)O3)=C3C=C2)=COC4=C1C=CC(O)=C4	SK–221	338.36	79.90
**Daidzein**<br>O=C1C(C2=CC=C(O)C=C2)=COC3=C1C=CC(O)=C3	SK–222	254.24	70.67
**Fumitremorgin C**<br>O=C([C@]1([H])CCCN21)N3[C@@H](/C=C(C)\C)C4=C(C5=C(N4)C=C(OC)C=C5)C[C@@] 3([H])C2=O	SK–223	379.46	65.64

**Bisdethiobis(methylthio)gliotoxin**<br>O=C1N(C)[C@@](SC)(CO)C(N2[C@@]1(SC)CC3=CC=C[C@H](O)[C@@]23[H])=O	SK–<br>224	356.47	81.07
**Cyclo-(L-Pro-L-Met)**<br>O=C(N[C@@H]1CCSC)[C@](CCC2)([H])N2C1=O	SK–<br>225	228.32	49.41
**Cyclo-(L-Pro-L-Tyr)**<br>O=C(N[C@H]1CC2=CC=C(O)C=C2)[C@@](CCC3)([H])N3C1=O	SK–<br>226	260.26	69.64
**Cyclo-(L-Pro-L-Phe)**<br>O=C([C@H](CCC1)N1C2=O)N[C@H]2CC3=CC=CC=C3	SK–<br>227	244.29	49.41
**Penicitrinone A**<br>O=C1C=C2OC3=C4O[C@H](C)[C@@H](C)C4=C(C)C(O)=C3C5=C2C([C@H](C)[C@@H](C)O5)=C1C	SK–<br>228	380.44	68.91
**Penicitrinone B**<br>O=C1C=C2OC3=C4OC(C)=C(C)C4=C(C)C(O)=C3C5=C2C([C@H](C)[C@@H](C)O5)=C1C	SK–<br>229	378.42	72.81
**2,3,4-trimethyl-5,7-dihydroxy2,3-dihydrobenzofuran**<br>OC1=C(OC(C)C2C)C2=C(C)C(O)=C1	SK–<br>230	194.23	49.69
**Physcion**<br>O=C1C2=C(C=C(C)C=C2O)C(C3=CC(OC)=CC(O)=C13)=O	SK–<br>231	284.27	83.83
**Aspergiketal**<br>O=C([C@]12OCCC1)C3=C(CO2)C(O)=C(C)C(O)=C3	SK–<br>232	250.25	76.00
**Sinulolide I**<br>O=C(OCC)CCCCCCCC[C@@](C(C)=C1C)(O)OC1=O	SK–<br>233	312.41	72.84
**Territrem A**<br>O=C1C2(C)C3(O)CC4=C(C=C(C5=CC(OC)=C(OCO6)C6=C5)OC4=O)OC3(C)CCC2(O) C(C)(C)C=C1	SK–<br>234	510.54	124.67
**Territrem B**<br>O=C1[C@]2(C)[C@@]3(O)CC4=C(C=C(C5=CC(OC)=C(OC)C(OC)=C5)OC4=O)O[C@]<br>3(C)CC[C@@]2(O)C(C)(C)C=C1	SK–<br>235	526.58	124.67
**Territrem C**<br>O=C1[C@]2(C)[C@@]3(O)CC4=C(C=C(C5=CC(OC)=C(O)C(OC)=C5)OC4=O)O[C@]3<br>(C)CC[C@@]2(O)C(C)(C)C=C1	SK–<br>236	512.55	135.67
**Territrem D**<br>C[C@@]12CC[C@@]3([C@@]([C@]1(CC4=C(O2)C=C(OC4=O)C5=CC(=C(C(=C5)OC)OC)<br>O)O)(C(=O)C=CC3(C)C)C)O	SK–<br>237	512.55	135.67
**Cowabenzophenone A**<br>O=C(C1(C/C=C(C)/CC/C=C(C)\C)C2)[C@@]3(C(C4=CC=CC=C4)=O)C(C)(C)[C@]2([H])C[C@]<br>5([H])C(C)(C)[C@@H](C(C)=C)CC(C3=O)5C1=O	SK–<br>238	598.80	68.25
**Asperteramide**<br>O=C(C(CC1=CC=C(O)C(C/C=C(C)\C)=C1)=C2C3=CC=C(O)C=C3)NC2=O	SK–<br>239	363.41	90.39
**Terreinlactone B**<br>O=C1OCCC(/C=C/C)=C1	SK–<br>240	138.17	26.30
**15bβ-hydroxyl-5-*N*-acetylardeemin**<br>O=C1N2C([C@](C[C@]3(C(CC=C)(C)C)C4N(C(C)=O)C5=C3C=CC=C5)(O)N4C([C@H]2C)<br>=O)=NC6=C1C=CC=C6	SK–<br>241	498.58	95.74
**10-phenyl-[12]-cytochalasin Z17**	SK–<br>242	463.57	92.70

O=C(C/C=C(C)\C([C@@H](C)C/C=C/[C@H]1[C@H](O)C(C)=C(C)[C@H]2[C@H](CC3=CC=CC=C3)N4)=O)O[C@]12C4=O			
**Neoasterriquinone.**<br>O=C1C(O)=C(C2=C(C/C=C(C)\C)NC3=C2C=CC=C3)C(C(O)=C1C4=C(C/C=C(C)\C)NC5=C4C=CC=C5)=O	SK–243	506.60	106.18
**(*3R*)-6,7,8-trihydroxymellein**<br>C[C@H]1OC(C2=C(C=C(O)C(O)=C2O)C1)=O	SK–244	210.19	86.99
**(*3R*)-8-methoxy-6-hydroxymellein**<br>C[C@H]1OC(C2=C(C=C(O)C=C2OC)C1)=O	SK–245	208.21	55.77
**4-chloro-3,6-dihydroxytoluquinone**<br>O=C1C(O)=C(Cl)C(C(C)=C1O)=O	SK–246	188.57	74.60
**6-hydroxytoluquinol hydrate**<br>OC1=C(C)C([C@@H](O)C[C@@H]1O)=O	SK–247	158.15	77.75
**Bisdethiobis(methylsulfanyl)acetylaranotin**<br>CC(O[C@@H](C=COC=C1C[C@@]23SC)[C@@]1([H])N3C([C@]4(SC)CC5=COC=C[C@H](OC(C)=O)[C@@]5([H])N4C2=O)=O)=O	SK–248	534.61	111.70
**Bisdethiobis(methylsulfanyl)acetylapoaranotin**<br>O=C1N2[C@](CC3=COC=C[C@H](OC(C)=O)[C@]32[H])(SC)CN4[C@@]1(SC)CC5=CC=C[C@H](OC(C)=O)[C@@]45[H]	SK–249	504.63	85.39
**Bisdethiobis(methylsulfanyl)apoaranotin**<br>O=C1N2[C@](CC3=COC=C[C@H](OC(C)=O)[C@]32[H])(SC)CN4[C@@]1(SC)CC5=CC=C[C@H](O)[C@@]45[H]	SK–250	462.59	79.31
**Asperbutenolide A**<br>CC(=CCC1=C(C=CC(=C1)C[C@@]2(C(=C(C(=O)O2)OC(C)(C)[C@H](CC3=C(C=CC(=C3)C[C@@]4(C(=C(C(=O)O4)O)C5=CC=C(C=C5)O)C(=O)OC)O)O)C6=CC=C(C=C6)O)C(=O)OC)O)C	SK–251	864.90	235.82
**Asperbutenolide B**<br>O=C([C@](C(C1=CC=C(O)C=C1)=C2O)(CC3=CC=C(O[C@H](C(C)(O)C)[C@H]4OC)C4=C3) OC2=O)OC	SK–252	470.47	131.76
**Asperbutenolide C**<br>O=C([C@](C(C1=CC=C(O)C=C1)=C2O)(CC3=CC=C(O[C@@H](C(C)(O)C)[C@@H]4OC)C4=C3)OC2=O)OC	SK–253	470.47	131.76
**Asperbutenolide D**<br>O=C(OC)C(CC1=CC=C(O)C(C/C=C(C)\C)=C1)(O)C(C2=CC=C(O)C=C2)=O	SK–254	384.43	104.06
**Asperbutenolide E**<br>O=C([C@](C(C1=CC=C(O)C=C1)=C2O)(CC3=CC=C(O)C(CC4OC4(C)C)=C3)OC2=O)OCC	SK–255	454.48	125.82
**Asperbutenolide F**<br>O=C(C(C(C1=CC=C(O)C=C1)=C2O)(CC3=CC=CC=C3)OC2=O)OCC	SK–256	354.36	93.07
**(*S*)-Methyl-4-hydroxy-2-[4- hydroxy-3-(3-methyl-but-2-enyl)- benzyl]-3-(4-hydroxy-phenyl)-5- oxo-2,5-dihydro-furan-2- carboxylic acid methyl ester**<br>O=C(O[C@]1(CC2=CC(C/C=C(C)/C)=C(O)C=C2)C(OC)=O)C(O)=C1C3=CC=C(O)C=C3	SK–257	424.45	113.29
**(*Z*/*E*)-L-phenylalaninamide**<br>O=C(C(N(C(C(NC(C)=O)C(C)C)=O)C)CC1=CC=CC=C1)N/C=C/C2=CNC3=CC=CC=C32	SK–258	460.58	94.30
**Furanaspermeroterpene A**	SK–259	558.58	159.59

O=C(OC)C[C@@]1([C@](OC2=O)(C(C)=O)O[C@@]3(O)C(C4C5(C)C)=O)[C@@]2(C)C([C@@H](OC(C)=O)C([C@@]13C)[C@]4(C)C=CC5=O)=C			
**Furanaspermeroterpene B** O=C(OC)C[C@@]1([C@](OC2=O)(C(C)=O)O[C@@]3(O)C(C4C5(C)C)=O)[C@@]2(C)C([C@@H](OC(C)=O)C([C@@]13C)[C@]4(C)CCC5=O)=C	SK–260	560.60	159.59
**Furanaspermeroterpene D** O=C(OC)C[C@@]1([C@]2(C)C([C@@]3(C)C4C(C)(C)C(C=C3)=O)[C@H](OC(C)=O)C([C@]15C)=C)C(OC2C4=O)=C(C)OC5=O	SK–261	526.58	122.29
**Furanaspermeroterpene E** O=C(OC)C[C@@]1([C@]2(C)C([C@@]3(C)C4C(C)(C)C(C[C@H]3OC)=O)[C@H](OC(C)=O)C([C@]15C)=C)C(O[C@@]2(O)C4=O)=C(C)OC5=O	SK–262	574.62	151.75
**Furanaspermeroterpene F** O=C(OC)C[C@@]1([C@](C(C(O)=C2C(C)(C)C(CC[C@]23C)=O)=O)(C)C3[C@H](O)C([C@]14C)=C)C([C@@H](C)OC4=O)=O	SK–263	502.56	144.28
**Furanaspermeroterpene G** O=C(OC)C[C@@]1([C@](C(C(O)=C2C(C)(C)C(C[C@@H](OC)[C@]23C)=O)=O)(C)C3[C@H](OC(C)=O)C([C@]14C)=C)C([C@@H](C)OC4=O)=O	SK–264	574.62	159.59
**Furanaspermeroterpene H** O=C(OC)C[C@@]1([C@](C(C(O)=C2C(C)(C)C(C[C@@H](OCC)[C@]23C)=O)=O)(C)C3[C@H](OC(C)=O)C([C@]14C)=C)C([C@@H](C)OC4=O)=O	SK–265	588.65	159.59
